# Systems analysis of non-parenchymal cell modulation of liver repair across multiple regeneration modes

**DOI:** 10.1186/s12918-015-0220-9

**Published:** 2015-10-22

**Authors:** Daniel Cook, Babatunde A. Ogunnaike, Rajanikanth Vadigepalli

**Affiliations:** Department of Chemical and Biomolecular Engineering, University of Delaware, Newark, DE USA; Daniel Baugh Institute for Functional Genomics/Computational Biology, Department of Cell and Developmental Biology, Thomas Jefferson University, Philadelphia, PA USA

**Keywords:** Liver regeneration, Molecular regulatory mechanisms, Systems biology, Regeneration modes, Chronic liver disease

## Abstract

**Background:**

A hallmark of chronic liver disease is the impairment of the liver’s innate regenerative ability. In this work we use a computational approach to unravel the principles underlying control of liver repair following an acute physiological challenge.

**Methods:**

We used a mathematical model of inter- and intra-cellular interactions during liver regeneration to infer key molecular factors underlying the dysregulation of multiple regeneration modes, including delayed, suppressed, and enhanced regeneration. We used model analysis techniques to identify organizational principles governing the cellular regulation of liver regeneration. We fit our model to several published data sets of deficient regeneration in rats and healthy regeneration in humans, rats, and mice to predict differences in molecular regulation in disease states and across species.

**Results:**

Analysis of the computational model pointed to an important balance involving inflammatory signals and growth factors, largely produced by Kupffer cells and hepatic stellate cells, respectively. Our model analysis results also indicated an organizational principle of molecular regulation whereby production rate of molecules acted to induce coarse-grained control of signaling levels while degradation rate acted to induce fine-tuning control. We used this computational framework to investigate hypotheses concerning molecular regulation of regeneration across species and in several chronic disease states in rats, including fructose-induced steatohepatitis, alcoholic steatohepatitis, toxin-induced cirrhosis, and toxin-induced diabetes. Our results indicate that altered non-parenchymal cell activation is sufficient to explain deficient regeneration caused by multiple disease states. We also investigated liver regeneration across mammalian species. Our results suggest that non-invasive measures of liver regeneration taken at 30 days following resection could differentiate between several hypotheses about how human liver regeneration differs from rat regeneration.

**Conclusions:**

Overall, our results provide a new computational platform integrating a wide range of experimental information, with broader utility in exploring the dynamic patterns of liver regeneration across species and over multiple chronic diseases.

**Electronic supplementary material:**

The online version of this article (doi:10.1186/s12918-015-0220-9) contains supplementary material, which is available to authorized users.

## Background

The liver’s unique ability to regenerate allows partial liver resection to be a viable treatment option for patients with various liver diseases. Because the liver can regenerate even when most of its mass is lost (up to ~75 %), typical treatment for hepatocellular carcinoma involves resection of liver mass containing tumors. Patients with liver diseases such as cirrhosis can be treated similarly using partial liver transplant from a live donor, followed by liver regeneration in both donor and recipient. Regenerative ability is not equal in all livers, however. Surgeons have long been wary of transplanting fatty livers from organ donors because fatty livers regenerate insufficiently or not at all [1]. Age, diet, and miRNA regulation have also been linked to the liver’s overall regeneration ability [2–6]. Additionally, many chronic diseases impair liver regeneration and repair following hepatic resection [7]. It has even been postulated that inhibition of the liver’s natural repair ability contributes to the progress of steatohepatitis to cirrhosis in the liver [8].

**Fig. 1:**
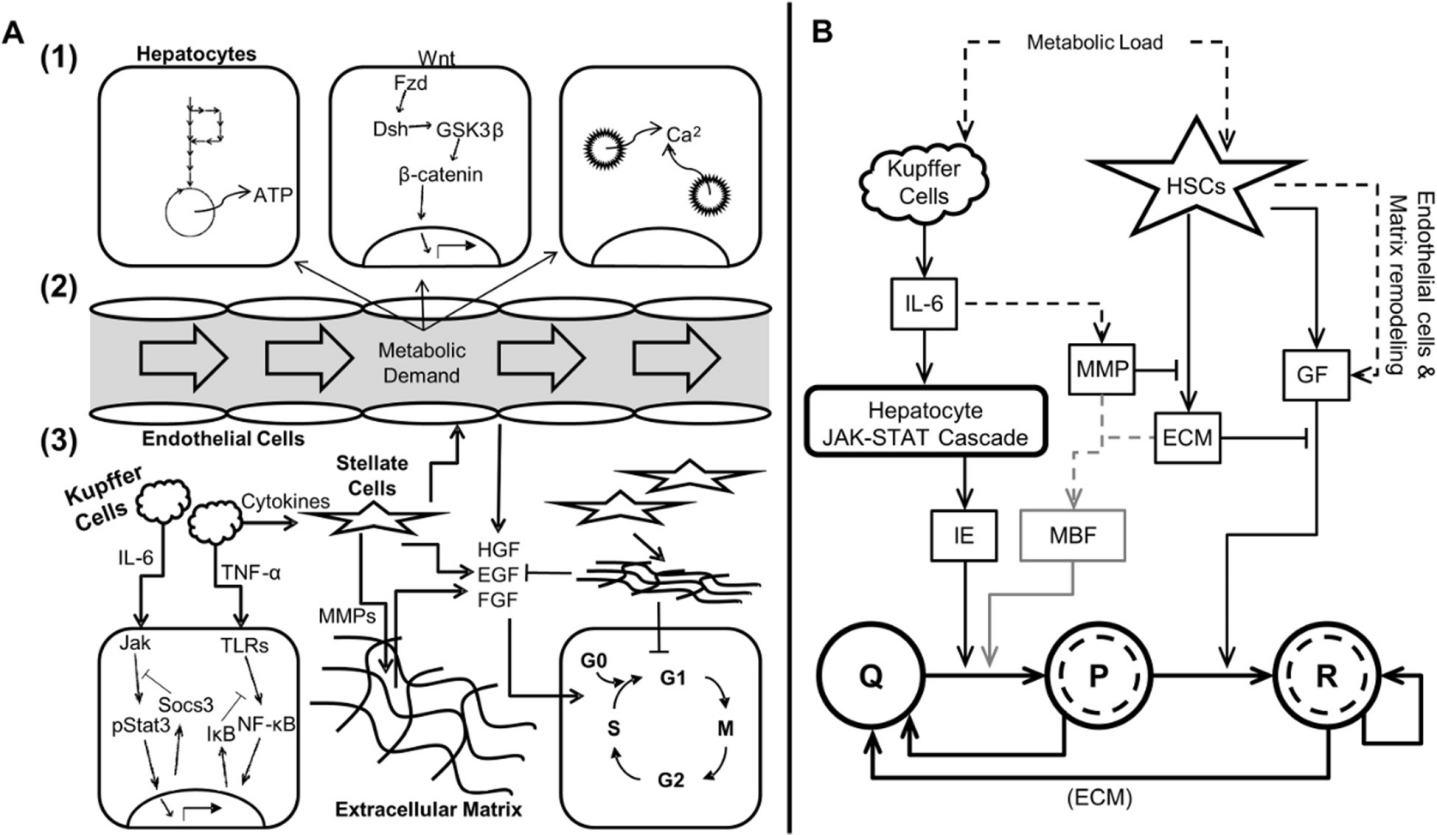
Schematic representation of the changes occurring during liver regeneration following PHx. **a** Detailed schematic. (1) Following PHx, hepatocytes respond within 30 s of tissue damage. Early post-PHx, previous work has shown release of ATP, increases in WNT signaling, and ionic Calcium release from hepatocyte mitochondria. (2) These responses in hepatocytes are likely to be driven by an increase portal blood flow, an increase in portal pressure, and an increase in metabolic demand per cell (increased nutrient availability, increased toxin flux, and increased extra-hepatic signals including LPS). (3) Signals from the blood and from hepatocytes activate non-parenchymal cells to produce factors governing hepatocyte entry into the cell cycle (including priming). **b** Simplified schematic diagram. This schematic shows the relationships included in the computational model. Several important pathways are lumped or represented as physiological transitions rather than including truly mechanistic detail. This physiological approach allows for insight into control principles of regeneration governed by archetypal signaling pathways. The gray matrix-bound factor (MBF) signaling was added to the model to investigate the contribution to liver mass recovery of matrix-bound signaling, but because of a relatively small impact on the dynamic mass recovery was excluded from further analyses

The mechanisms governing liver regeneration have been studied extensively over the last several decades (Fig. 1a) [9–11]. In summary, following partial hepatectomy (PHx), hepatocytes respond within 30 s of tissue damage. This early hepatocyte response consists of multiple signals, including release of ATP, increases in WNT signaling, and ionic calcium release from hepatocytes. These responses in hepatocytes are likely driven by extrinsic factors including an increase in portal blood flow, an increase in portal pressure, an increase in metabolic demand per cell (increased nutrient availability, increased toxin flux, and increased extra-hepatic signals including LPS), and signaling from factors liberated from the extracellular matrix coupled with intrinsic factors including hepatocyte metabolic capacity, functional history, and transcriptional state. Signals from the blood and from hepatocytes activate non-parenchymal cells to produce factors governing hepatocyte entry into the cell cycle. Kupffer cells respond within the first hour post-PHx to produce cytokines and chemokines that signal to hepatocytes through the JAK-STAT3 and NF-κB pathways and prime hepatocytes for replication. These cytokine signals, coupled with hepatocyte and blood signals, also activate hepatic stellate cells and endothelial cells to produce growth factors directly, through *de novo* synthesis, and indirectly, through matrix remodeling and release of matrix-bound growth factors. These growth factors induce hepatocytes to enter the cell cycle. While liver mass is still low, non-parenchymal cells maintain high growth factor bioavailability, which maintains hepatocytes in the cell cycle as liver mass regenerates. Following recovery of liver mass, the termination stage of regeneration begins. During the termination stage, hepatocytes exit the cell cycle and re-enter the G0 phase. This requiescence is thought to be governed by a combination of accumulation of extracellular matrix, requiescence of non-parenchymal cells, and an alteration of hepatocyte transcriptional programs, for example the renormalization of the C/EBP-α and C/EBP-β switch [12].

Despite clinical relevance and advances in our understanding of the molecular mechanisms underlying regeneration, however, the organizational principles governing molecular regulation of liver regeneration remain unclear. To investigate these organizational principles, a computational model of liver regeneration was developed recently, taking into account growth factor (GF) signaling, cytokine signaling along the JAK-STAT pathway, and hepatocyte replication [13]. Furchtgott, Chow, and Periwal employed this computational model to account for differential regeneration profiles after various degrees of partial hepatectomy. This model considered cell proliferation but not cell growth, thus limiting its ability to account for liver repair scenarios that involve hypertrophy in addition to hyperplasia.

In this study, we address this issue by extending the cell phenotype based computational model of liver regeneration to include both cell growth and replication (represented schematically in Fig. 1b). We employ this extended model to investigate quantitatively how altering the molecular regulation of hepatocytes affects the liver’s innate repair ability. Our extended model maintains the structure of the original model by combining classes of molecular signals with physiological observations of regeneration to capture dynamic regeneration phenotypes. The parameters and variables included in our model as well as their approximate biological correlates are provided in Table 1. Briefly, in our model, hepatocytes can exist in one of three states: Quiescent (Q), Primed (P), or Replicating (R). An increased metabolic load (metabolic demand per cell or M/N) induced by partial hepatectomy serves as the initial signal inducing regeneration in the model (Additional file 1: Figure S1). Our model uses a lumped approach to modeling these initiating signals by combining both extrinsic and intrinsic factors into the metabolic demand parameter. This increased metabolic load is modeled as directly activating non-parenchymal cells, specifically Kupffer cells (KC) and hepatic stellate cells (HSC), although this activation likely also involves signals from hepatocytes. Inflammatory cytokines produced predominantly by Kupffer cells (represented in the model by IL-6) signal through the JAK-STAT signaling pathway in hepatocytes to induce production of immediate early (IE) genes, which induce hepatocytes to enter the Primed state. Primed hepatocytes are able to respond to GF signaling from hepatic stellate cells and enter the Replicating state. Once in the Replicating state, hepatocytes maintain replication until natural requiescence or buildup of extracellular matrix (ECM) induces them back to the Quiescent state. The main benefit provided by our extended model is that hepatocytes that respond to the increased metabolic load (P and R) can increase their mass to meet metabolic load as well as enter replication. This corresponds to the biological scenarios of a cell increasing its mass prior to replication or becoming larger to meet a functional demand.

**Table 1:** Model variables and parameters and their approximate biological correlates

Parameters		
Name	Nominal or Starting Value	Approximate Biological Correlate
M	20.8 (rat)	Relative nutrient and toxin delivery/absorption rate in the liver
G	3.5x10^−4^ (rat)	Growth rate of hepatocyte mass [mass equivalent doublings/min]
k_IL6_	1.5	Rate at which non-parenchymal cells (primarily Kupffer cells) are able to modify the cytokine milieu post-PHx
κ_IL6_	0.9	Rate of cytokine degradation
V_JAK_	2x10^4^	Maximum JAK activation rate
K_m_^JAK^	10^4^	JAK Michaelis concentration
κ_JAK_	0.4	Rate of JAK degradation
[STAT3]	2	Relative concentration of monomeric STAT3 in the liver
V_STAT3_	7.5x10^2^	Maximum STAT3 phosphorylation rate
K_m_^STAT3^	0.4	pSTAT3 Michaelis concentration
κ_STAT3_	0.1	Rate of pSTAT3 dephosphorylation
V_SOCS3_	2.4x10^4^	Maximum SOCS3 activation rate
K_m_^SOCS3^	7x10^−4^	SOCS3 Michaelis concentration
κ_SOCS3_	0.4	Rate of SOCS3 degradation
K_I_^SOCS3^	1.5x10^−2^	SOCS3 Inhibition effect on STAT3 phosphorylation
V_IE_	2.5x10^2^	Maximum IE gene activation rate
K_m_^IE^	18	IE gene Michaelis concentration
κ_IE_	5	Rate of IE gene degradation
κ_DEG_	7	Rate of ECM degradation by MMPs
κ_ECM_	33	Rate of constitutive ECM degradation
k_GF_	0.113	Rate at which non-parenchymal cells (primarily hepatic stellate cells) directly & indirectly produce growth factors post-PHx
κ_GF_	0.23	Rate of growth factor degradation
k_up_	6x10^−2^	Rate of growth factor absorption/binding to the ECM
k_Q_	7x10^−3^	Maximum rate of hepatocyte transition from Quiescence to Primed [cells/min]
k_P_	4.4x10^−3^	Maximum rate of hepatocyte transition from Primed to Replicating [cells/min]
k_R_	5.4x10^−3^	Maximum rate of hepatocyte transition from Replicating to Quiescence [cells/min]
k_prol_	2x10^−2^	Rate of hepatocyte progression through the cell cycle [doublings/min]
k_req_	0.1	Requiescence rate of Primed hepatocytes [cells/min]
θ_req_	8	None
β_req_	3	None
k_ap_	0.1	Apoptosis rate of damaged hepatocytes
θ_ap_	9x10^−3^	None
β_ap_	4.5x10^−3^	None
k_MBF_	1	Rate of release of matrix bound factors during ECM remodeling
κ_MBF_	1	Degradation rate of matrix bound factors once they are released from the ECM
Variables		
Name	Nominal or Starting Value	Approximate Biological Correlate
Q	1	Fraction of hepatocytes in the Quiescent state
P	0	Fraction of hepatocytes in the Primed state
R	0	Fraction of hepatocytes in the Replicating state
[IL-6]	1	Cytokine microenvironment of the liver
[JAK]	1	Relative levels of activated receptors for cytokine signals in hepatocytes
[pSTAT3]	1	Relative levels of phosphorylated STAT-3 compared to monomeric STAT-3 or other downstream effectors of cytokine signaling (i.e. NF-κB)
[SOCS3]	1	Relative levels of SOCS3 or other inhibitors of cytokine signaling
[IE]	1	Relative levels of immediate early genes induced in hepatocytes (e.g. cFOS, cJUN, and AP-1)
[GF]	1	Relative bioavailability of growth factors promoting hepatocyte proliferation
[ECM]	1	Relative levels of extracellular matrix buildup of matrix composed of collagens inhibitory to regeneration
[MBF_ECM_]	50	Relative levels of matrix bound factors priming hepatocytes
[MBF_Free_]	0	Relative levels of free matrix bound factors that were initially bound by ECM

Additionally, we extended the model further to consider explicitly the contributions of initially matrix-bound factors, MBFs, (including growth factors and potentially WNT precursors), that are liberated from the matrix during remodeling post-PHx. These factors likely contribute to the quiescent-to-primed transition that hepatocytes undergo during the priming phase and may be equally as important as the early, predominantly Kupffer cell-produced cytokine microenvironment in priming hepatocytes for entry to the cell cycle (Fig. 1b, gray portion).

Our extended computational model allowed us to investigate several issues outstanding in the field of liver regeneration. We analyzed the extended model to determine the relative contributions of the predominantly Kupffer cell-produced cytokine microenvironment and the ECM-liberated signals to prime hepatocytes to enter the cell cycle. We simulated the extended model over a wide range of parameter values and identified parameter sets giving rise to distinct modes of liver regeneration and common or unique molecular regulation of liver regeneration dynamics. We next questioned what organizational principles regulate the biology of liver regeneration. Our model-based analyses revealed how altering regulatory balances can shift the liver into distinct, clinically relevant regeneration modes. We analyzed multiple published regeneration profiles to identify common organizational principles underlying liver regeneration across distinct tissue response phenotypes. We then predicted which molecular signaling dysregulation may account for altered liver regeneration profiles in multiple species and disease scenarios. We also used our model to compare several hypotheses about differences in regeneration between humans and rats, and suggest measurements that can be used to test these hypotheses. We hope that understanding how organizational principles work together to govern the dynamics of liver regeneration and repair will provide unique insights into liver disease progression, suggest further avenues of research for targeted therapy for chronic liver diseases, and provide insights into treatments to promote liver regeneration after surgical resection.

## Results

### Model implementation

Our computational model extends the model previously published by Furchtgott, Chow, and Periwal by adding terms describing the contributions of cell growth and initially matrix-bound factors to liver regeneration following resection [13]. Our computational model consists of 11 ODEs (described in detail in the Methods section), 43 parameters (Table 1), and 12 variables representing molecular levels and cell abundances (Table 1). The Matlab code used for this study is available as supplemental information in Additional file 2. All variables representing molecular levels, except matrix bound factors (MBF), have an initial steady-state level of 1 and any change thereafter is a fold-change over baseline. Determination of MBF initial level is described in the following section. The initial level of quiescent hepatocytes is 1, while initial levels of primed and replicating hepatocytes are 0. All simulations were performed using Matlab (Mathworks, Natick, MA).

### Extended model predicts the importance of Kupffer cell-mediated signaling during the priming phase

The importance of direct intercellular signaling leading to IE gene expression has been widely studied. Direct interventions to intercellular signaling have been shown to impact liver regeneration dynamics significantly [14]. Whereas, the effects of matrix bound factors (MBF) are less well appreciated but appear to have a more subtle effect on regeneration dynamics [15]. Therefore, we reasoned that the effects of MBF are likely less than the effects of IE genes on driving regeneration. We tested model behavior if the effects of MBFs are just as important to regeneration as IE gene effects. Rather than match parameters for MBF signaling to a particular MBF (i.e. WNT) we tuned the model parameters initial MBF levels, production rate, and degradation rate such that the relative magnitude of the priming signal from initially MBF signaling and IE gene production were of the same order of magnitude during the timeframes when they were contributing to hepatocyte priming (Additional file 3: Figure S2). Table 1 contains the parameters that correspond to this phenotypic behavior. This parameter choice relies on the assumption that MBF signaling is as important as IE gene production to induce hepatocyte priming, and MBFs are depleted following the priming phase. Unbinding of MBF peaked approximately 45 min post-PHx and lasted over the duration of the priming phase (6 h post-PHx), while IE gene levels peaked close to 3 h post-PHx and remained high throughout the early stages of liver regeneration (>12 h post-PHx). We found that including MBF signaling altered the dynamic mass recovery only slightly, leading to a sustained offset in mass recovery compared to the case without MBF signaling (Additional file 4: Figure S3). The effect of MBF signaling in our model is slight most likely because the duration of MBF signaling is shorter than the duration of cytokine signaling. Because of the negligible effect that MBF signaling had on liver regeneration dynamics, we excluded its contributions from the subsequent model analyses.

### Extended model with cell growth better accounts for rat liver regeneration profile

**Fig. 2:**
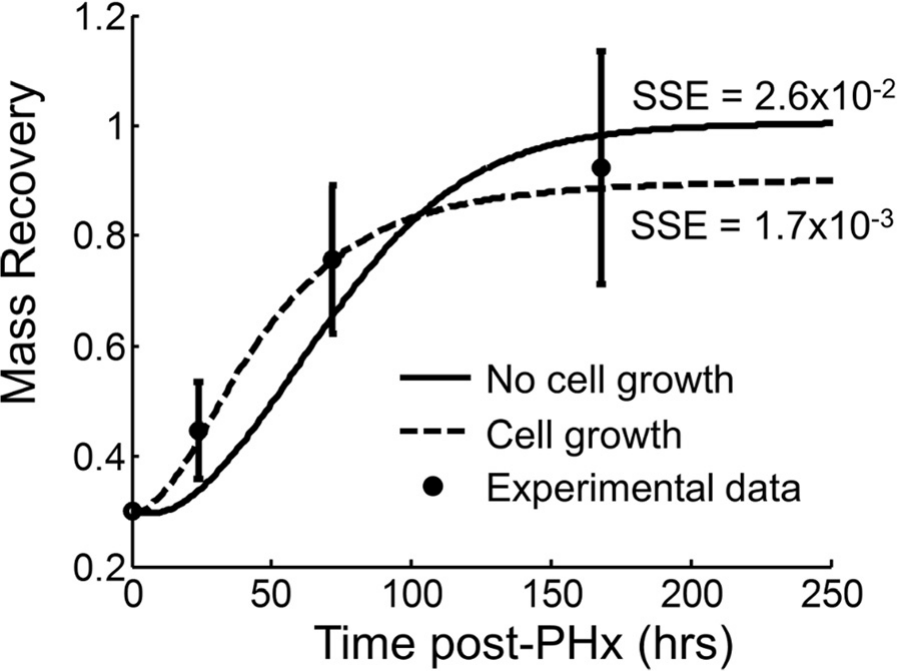
Comparison of experimental data of liver regeneration in rats from Tanoue et al. [16] (Experimental data) to the models proposed by Furchtgott et al. [13] (No cell growth) and proposed in this work (Cell growth). The model proposed in this work is able to fully capture the dynamics of liver repair following 70 % partial hepatectomy in rats

Although the original model proposed by Furchtgott et al. [13] captured the broad features of liver regeneration in rats, it considered relative hepatocyte number as a measure of tissue response rather than overall mass. Comparing this simulated number of hepatocytes to experimental data is difficult because the experimentally available measurement closest to cell number is relative tissue mass. When compared to relative tissue mass recovery, this model fails to match the mass recovery dynamics accurately: specifically, the model without cell growth fails to capture the experimental observation that the rat liver doubles in mass by 24 h post hepatectomy (Fig. 2, “No cell growth” & “Experimental data”) [13, 16]. Our extended model incorporating cell growth could better account for the dynamic profile of liver mass recovery in rats by more accurately simulating mass recovery dynamics (Fig. 2, “Cell growth”). We performed a log-likelihood ratio test to assess whether our extended model described the experimental data significantly better than the previous model. This test takes into account the number of parameters used in the model and the model error in fitting the experimental data. We assumed that the residuals from the fitted models followed a Gaussian distribution (i.e. there was no non-random pattern to the residuals) and used one degree of freedom, corresponding to the cell growth parameter we added to the model. For further explanation of the test, see the Methods section. We found that the original model had a log-likelihood of 4.42, while our extended model had a log-likelihood of 9.64. The results of log-likelihood ratio test showed that our extended model was able to capture the experimental data more accurately than the previous model, with a *p*-value of 0.0012 (G^2^ = 10.53). The ability to compare our simulated regeneration profiles to experimental mass recovery profiles allowed us to simulate experimentally observed cases of deficient liver regeneration and predict molecular and physiological deficiencies underlying these cases.

### Exploring the state space of liver regeneration reveals distinct regeneration modes

**Fig. 3:**
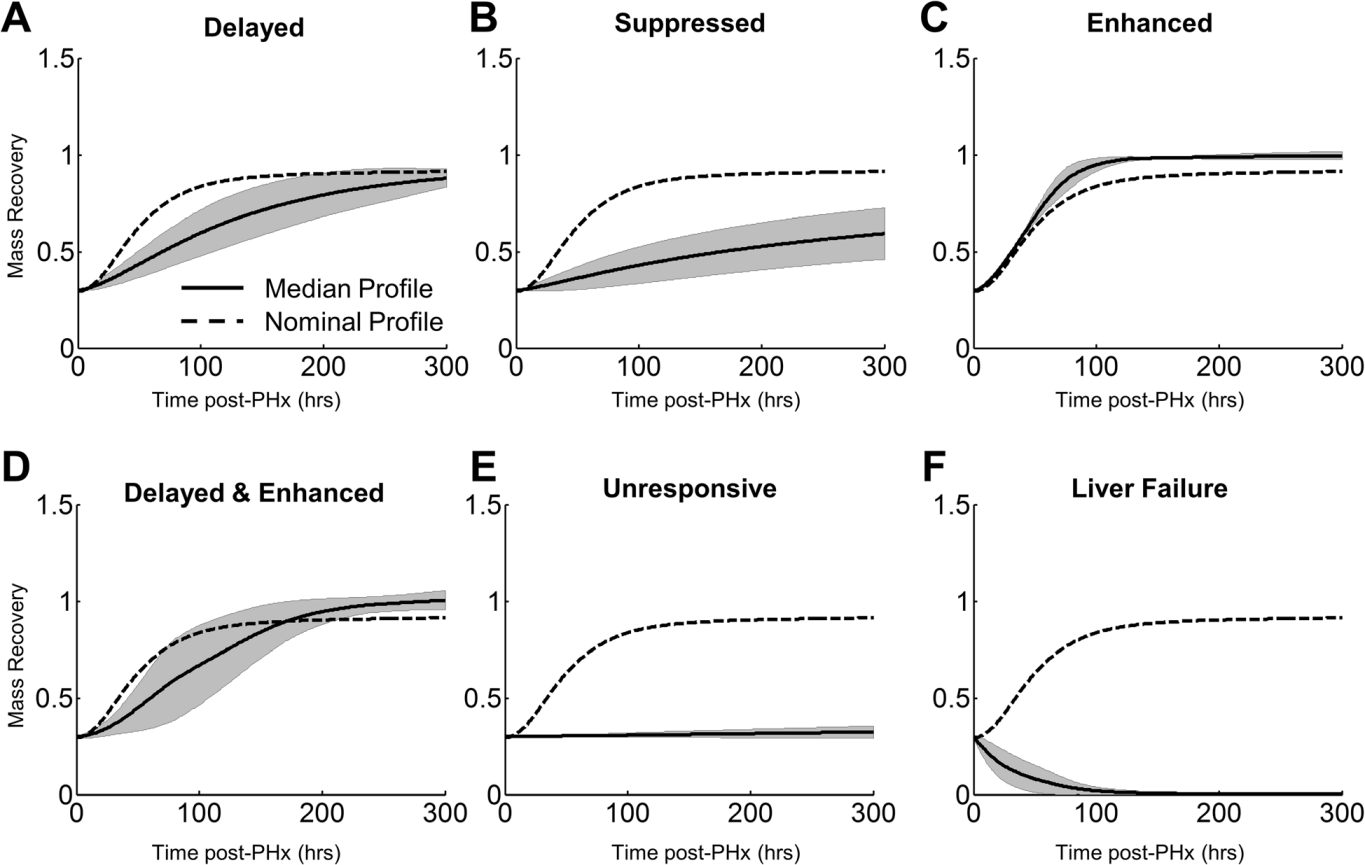
Model-predicted modes of regeneration revealed through sampling model parameters (+/− 50 % of nominal values). Varying model parameters simultaneously revealed distinct regeneration modes, including (**a**) delayed, (**b**) suppressed, (**c**) enhanced, (**d**) delayed and enhanced, (**e**) unresponsive, and (**f**) liver failure. The dashed line is the nominal profile; the gray areas indicate +/− 1 standard deviation

We sampled the model’s parameter space within a range of biologically reasonable parameter values using a Latin hypercube sampling method to sample each parameter uniformly from +/− 50 % of its nominal value. We then simulated liver regeneration following 70 % PHx using 150 parameter sets and classified the resulting regeneration dynamics. We found that liver regeneration is classifiable into several distinct modes of response to PHx: four regenerating modes (Fig. 3a-d): delayed, suppressed, enhanced, and delayed and enhanced; and two non-regenerating modes (Fig. 3e,f): unresponsive and liver failure.

**Fig. 4:**
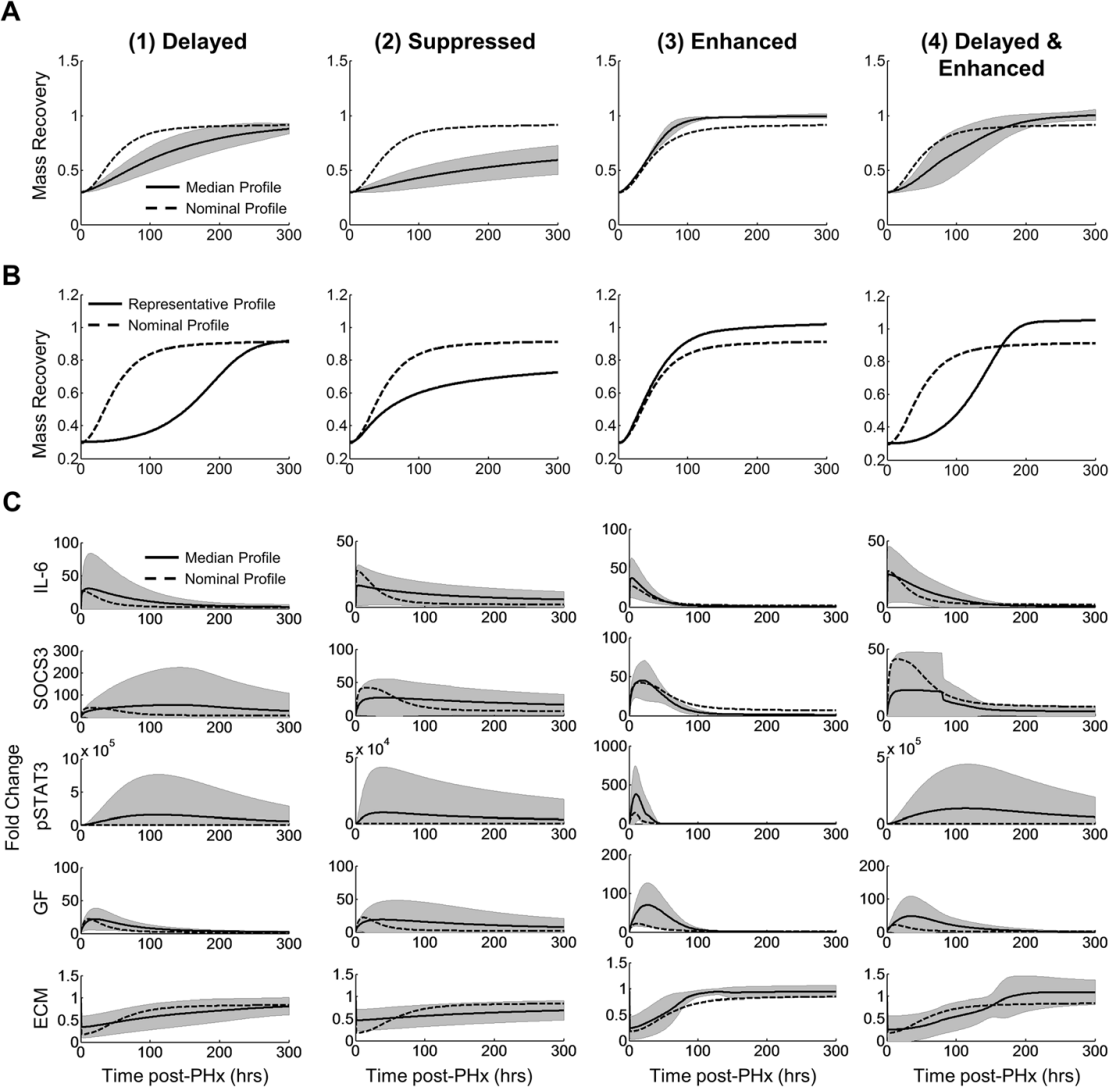
Molecular regulation governing altered regeneration profiles. **a** Average mass recovery, **b** One representative instance of mass recovery, **c** Average molecular regulation for regeneration modes: (1) Delayed, (2) Suppressed, (3) Enhanced, (4) Delayed and Enhanced. Dashed line represents nominal profile, black line represents average (or [B] one instance of the) profile, gray area represents +/− 1 standard deviation

Next, we investigated the molecular regulation governing the distinct regeneration modes (Fig. 4). We found that for most of the regeneration modes the variability in molecular regulation was high, often overlapping both the nominal regeneration case and zero levels (Fig. 4c). These results show that there is no single molecular profile that gives rise to a particular regeneration mode and that imbalances in a combination of factors can have large effects on regeneration dynamics. Based on these results, we conclude that the balance and timing of multiple factors acting in combination is critical in shaping the regeneration mode following resection. We further investigated specific molecular imbalances that could lead to instances of each altered regeneration modes. These investigations can be found in the supplemental figures (Additional file 5: Figure S4; Additional file 6: Figure S5; Additional file 7: Figure S6; Additional file 8: Figure S7; Additional file 9: Figure S8; Additional file 10: Figure S9).

### Sensitivity analysis reveals that molecular and physiological regulation strongly affects dynamic mass recovery

**Fig. 5:**
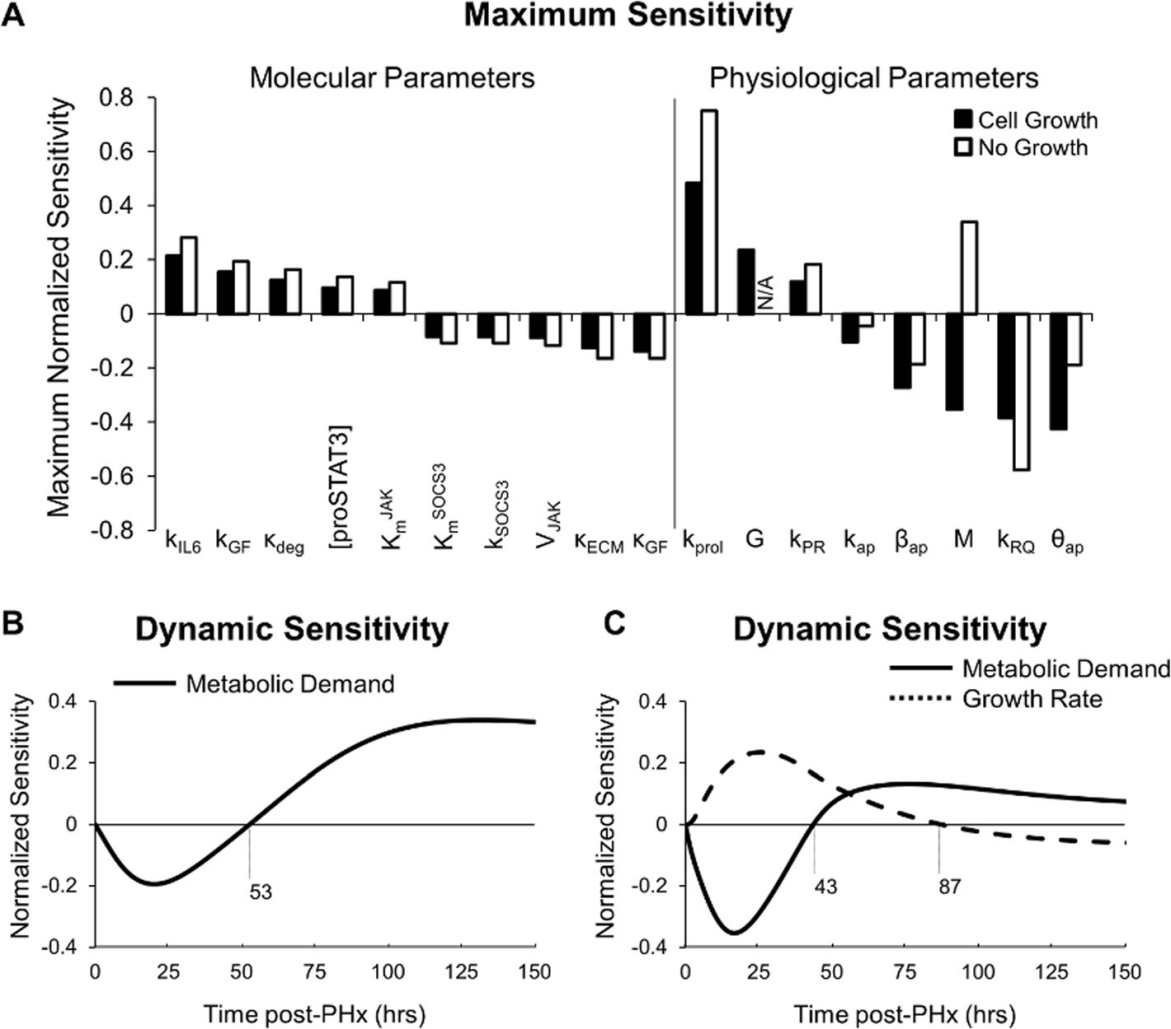
Dynamic local sensitivity analysis of the regeneration model with and without cell growth. **a** Maximum normalized sensitivities were calculated for each parameter. **b** Normalized local sensitivity of metabolic demand when not considering cell growth. Metabolic demand is inhibitory for the first 53 h post-PHx, likely through increases in cell apoptosis. After 53 h, metabolic demand enhances regeneration, likely through increased production of growth factors. **c** Normalized local sensitivities of metabolic load and growth rate reveal a dynamic competition between replication and growth. From 43–87 h post-PHx growth rate and metabolic demand both drive regeneration. During initiation and termination, however, imbalances between growth and metabolic load can inhibit regeneration

We performed a local parametric sensitivity analysis to identify additional factors and network balances significantly affecting the liver regeneration dynamics. We found that the addition of cell growth to the extended model did not have a strong effect on the maximum local sensitivity coefficients of model parameters (Fig. 5a). The exception to this observation is the maximum sensitivity of the metabolic demand parameter (M), which changed from positive to negative with the addition of cell growth. Sensitivity values computed for both the original model and the extended model including cell growth revealed that both molecular and physiological parameters showed high sensitivity. The model’s physiological parameters showed the highest absolute sensitivities, suggesting that such a lumped approach to studying tissue behavior may exclude detailed predictions about important biological processes. Model parameters related to production of factors from non-parenchymal cells as well as model parameters related to hepatocyte response to these factors showed high sensitivity, suggesting that parenchymal and non-parenchymal cell regulation are both important for governing liver regeneration. Specifically, we identified a potential antagonism between GF production rate (k_GF_) and degradation rate (κ_GF_) and between IL-6 production rate (k_IL-6_) and IL-6 degradation rate (κ_IL-6_) (Additional file 11: Figure S10). Increasing the production rates of IL-6 and GF enhanced overall regeneration, while increasing degradation rates inhibited overall regeneration.

We investigated how the inclusion of cell growth modified the dynamic sensitivity of the metabolic demand parameter (M). When cell growth is not considered, increased metabolic demand was inhibitory to liver recovery during the first 53 h post-PHx, largely due to increased hepatocyte apoptosis (Fig. 5b). After 53 h, increased metabolic demand enhanced regeneration. With cell growth considered, the initial inhibitory effect of increasing metabolic demand lasted only for the first 43 h post-PHx, after which it enhanced mass recovery but to a lower extent than the model without cell growth (Fig. 5c). The inclusion of cell growth also allowed us to recognize a potential dynamic antagonism between metabolic demand and cell growth rate. Early post-PHx, hepatocyte growth was a positive contributor to liver regeneration, while metabolic demand negatively affected progression of regeneration. At this early time, metabolic demand acted in hepatocytes predominantly to induce apoptosis in damaged cells through high metabolic load, causing reduced liver mass. After approximately 43 h post-PHx, high metabolic load induced high response in non-parenchymal cells causing increased priming and regeneration. From approximately 43 to 87 h post-PHx, metabolic load and hepatocyte growth acted synergistically to promote liver regeneration. Near the termination stage of liver regeneration, however, hepatocyte growth inhibited liver regeneration by inducing hepatomegaly and decreasing the driving force for regeneration.

### Paired parameter analysis reveals control principles governing the network balances driving liver regeneration

**Fig. 6:**
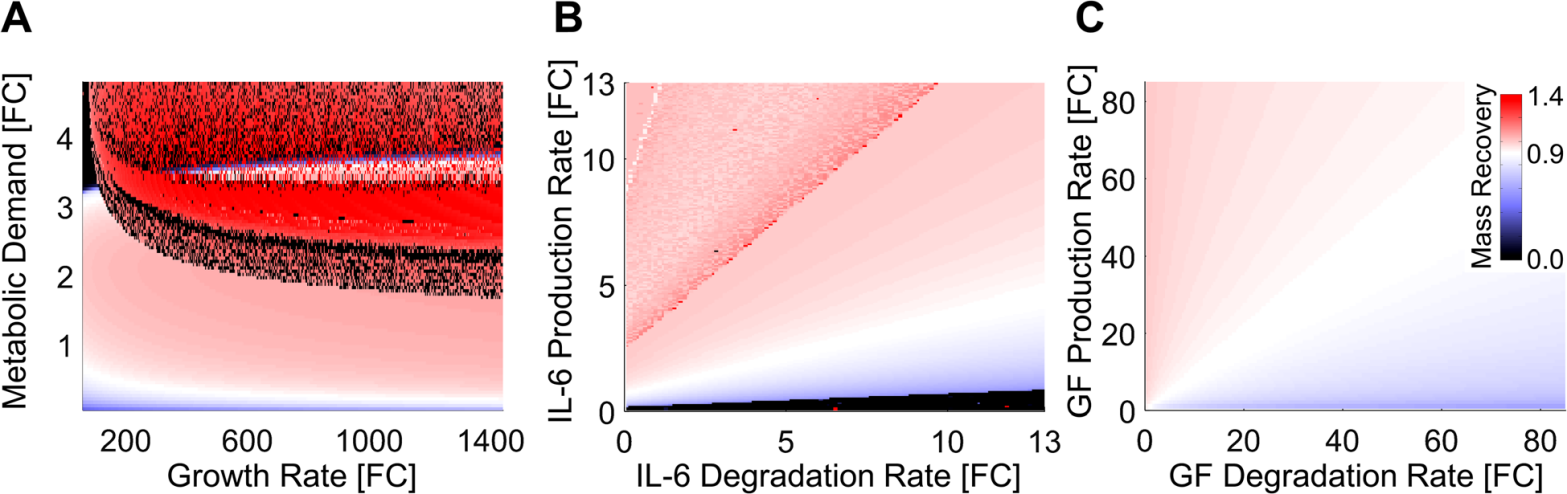
Heatmaps show the overall liver mass recovered is sensitive to combinatorial effects of **a** Metabolic load and hepatocyte growth rate, **b** IL-6 turnover rate, and **c** GF turnover rate. All parameter changes are displayed in Fold change [FC] over nominal parameter value. **a** The proper balance between regenerative drive and cell growth in response to stress is required for normal regeneration. **b** IL-6 and **c** GF production rates have large-scale effects on overall recovery, while degradation rates act as fine tuning

We investigated the organizational principles during liver regeneration by independently varying the pairs of antagonistic parameters identified from the sensitivity analysis. We varied each parameter over an order of magnitude and simulated overall liver mass recovery. We found that although increasing metabolic demand and hepatocyte growth rate had opposing effects during the beginning and end of liver mass recovery, simultaneously increasing these parameters tended to cause an increase in overall mass recovery (Fig. 6a). When changed together, metabolic demand has a much stronger effect on overall mass recovery than cell growth rate, for metabolic demand parameter values lower than approximately 40 (or an approximate fold change of 2). When metabolic demand was high (>2 fold change), regeneration is typically enhanced but certain growth rates coupled with these high metabolic demands could cause complete liver failure or govern the magnitude of the enhanced recovery. When metabolic demand was high (>3 fold change) and growth rate was low (<0.05, or approximately 200 fold change), growth rate was not able to compensate for increased apoptosis caused by high metabolic demand and liver failure occurred. Based on the results near nominal parameter values for metabolic demand and cell growth rate, metabolic demand leading to cell replication appears to be a primary driver of liver repair following damage, while cell growth may be a secondary or compensatory driver.

When we investigated the relationship between IL-6 and GF production and degradation, we found that relatively slight increases to both IL-6 (Fig. 6b) and GF (Fig. 6c) production rate increased mass recovery, while degradation had to increase much more to cause an equivalent magnitude decrease in mass recovery. This antagonism was more pronounced in IL-6 balance, but was relatively subtle in GF balance. For further visualization of the effects of GF production and degradation balance, see Additional file 12: Figure S11. These relationships reveal an organizational principle whereby production of molecules acts as a means of achieving coarse-grained control of molecular levels while degradation acts to achieve fine-tuned control. These results suggest that non-parenchymal cells may act predominantly as coarse-grained controllers of liver regeneration, while hepatocyte responsiveness and miRNA or other regulation may act to achieve fine-tuned control of liver regeneration.

**Fig. 7:**
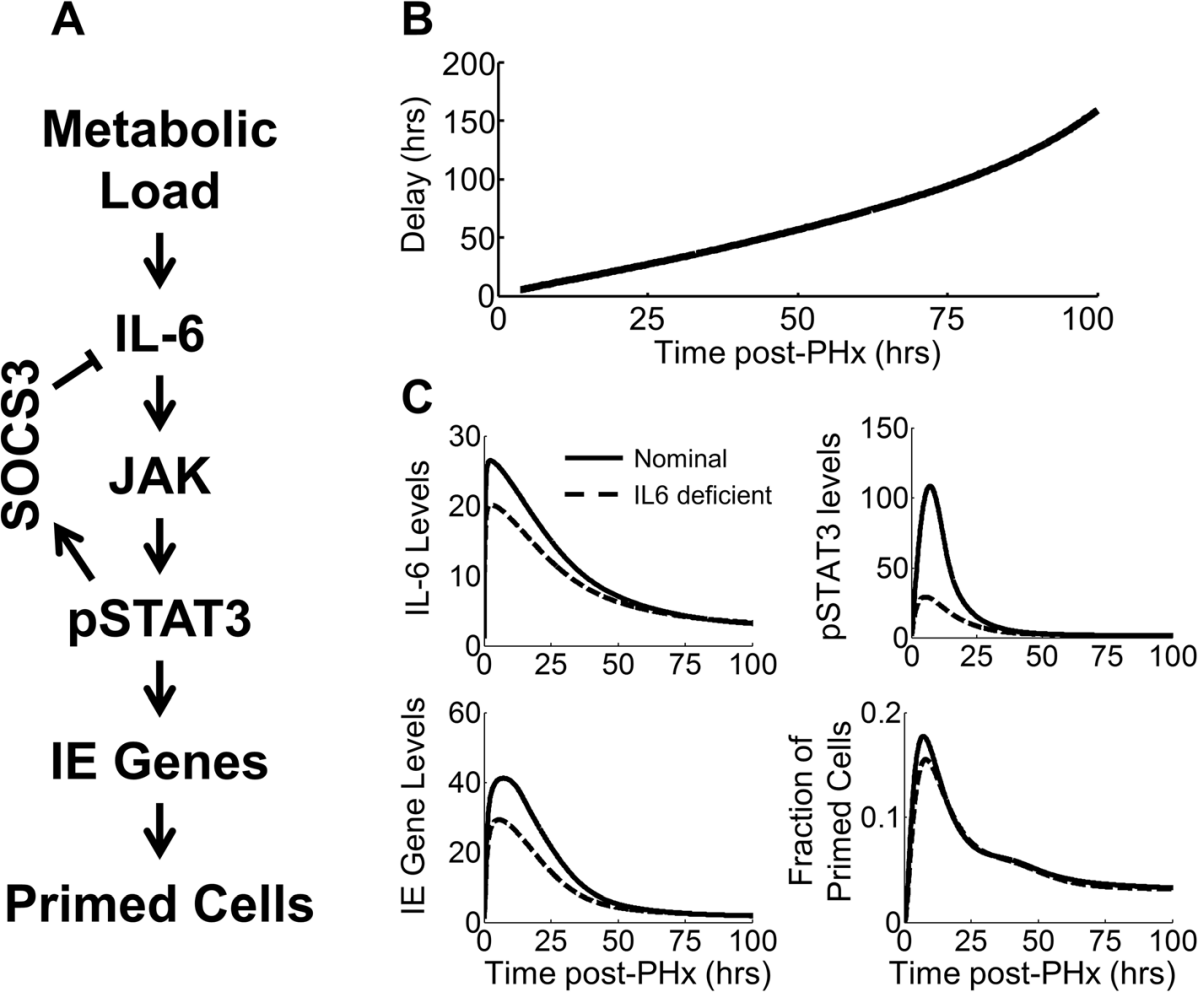
Effect of decreasing IL-6 production. **a** IL-6 signals through the JAK-STAT signaling pathway to prime hepatocytes for replication. **b** Reduced IL-6 production causes delayed regeneration, with delays increasing as regeneration progresses. **c** A slight decrease in IL-6 production (25 % reduction in peak levels) amplifies as the signal propagates through the JAK-STAT cascade. Ultimately, this slight IL-6 decrease results in reduced STAT-3 phosphorylation (75 % reduction in peak) and reduced priming (~10 % reduction at peak). This reduced priming leads to delayed recovery and a slightly reduced overall mass recovery (~5 %)

We further investigated the mechanisms through which cytokines and growth factors affect regeneration dynamics. We found that the immediate inflammatory response to partial hepatectomy, represented in the model by IL-6 signaling (Fig. 7a), controlled the timing of the regeneration response (Fig. 7b) by controlling the magnitude of the priming response (Fig. 7c). In simulations, a slight reduction in IL-6 production rate (causing an ~25 % decrease in peak IL-6 levels) led to significantly decreased STAT-3 phosphorylation (~75 % reduced) and a lower priming response (~10 % reduced) caused by decreased IE gene signaling (Fig. 7c). In addition, this decrease in IL-6 levels not only lowered the priming response (Fig. 7c) but also slightly delayed the peak of priming, from 7 to 8 h post-PHx. This early impaired priming response propagated through the time course of regeneration, lengthening the recovery for IL-6 signaling deficient cases (Fig. 7b). This result indicates that relatively small upstream events can have a substantial effect on overall recovery. It is important to consider, however, that many biological processes (including inflammatory molecule production and secretion, receptor binding, competition with anti-inflammatory molecules and signaling pathways, and cellular responsiveness to inflammation) contribute to the simulated IL-6 signaling. Deficiencies in any steps within these processes could lead to the deficient priming indicated by the model simulation.

**Fig. 8:**
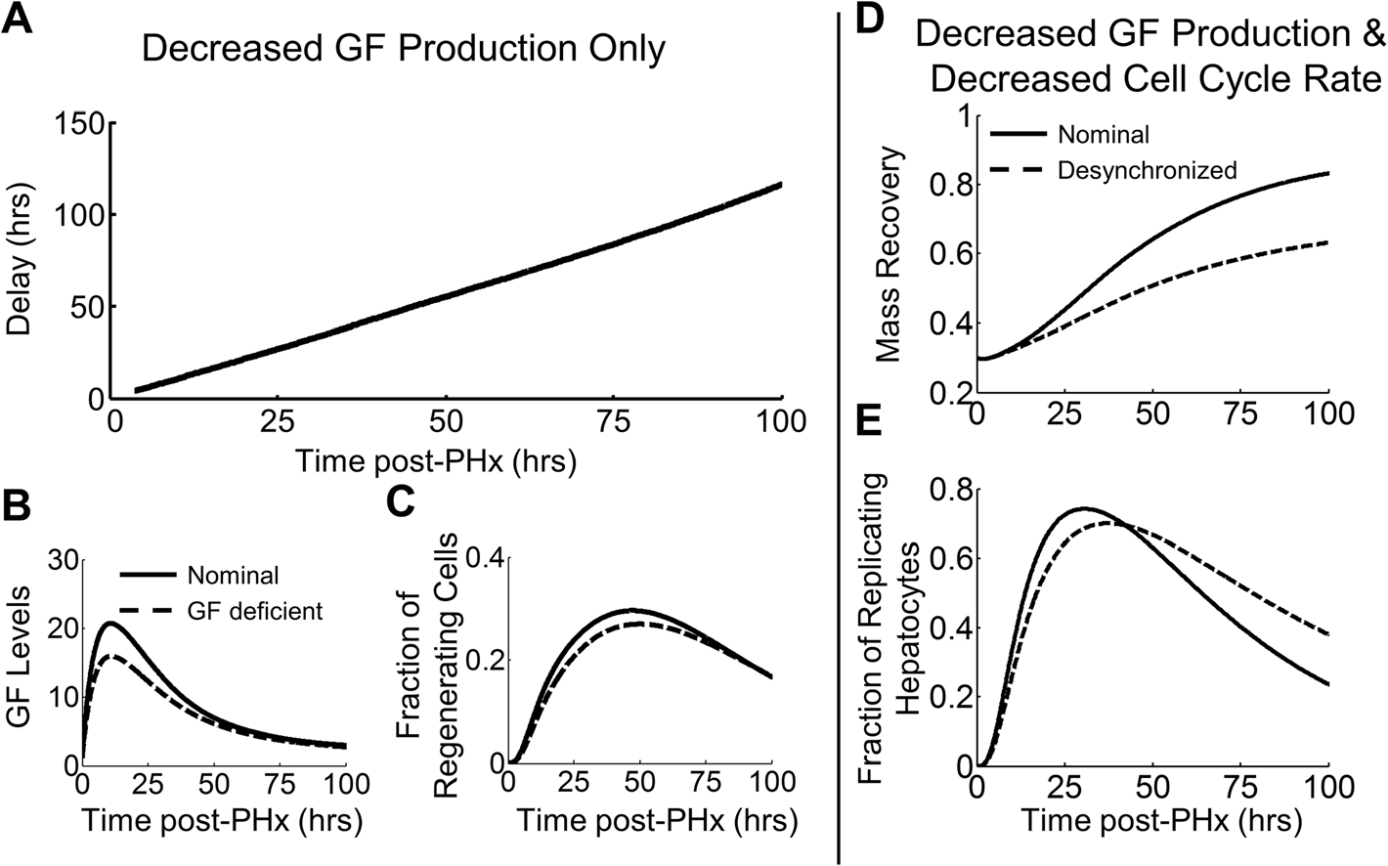
Effects of decreasing GF bioavailability. **a** Decreased GF production causes a delay in regeneration. **b** As GFs become less available, **c** fewer hepatocytes enter the cell cycle, decreasing peak of regenerating cells. The synchronicity of hepatocyte entry into the cell cycle, however, is affected only slightly. **d**-**e** To decrease the synchronicity of entry into the cell cycle, it is necessary to decrease the proliferation rate

Growth factor bioavailability, in contrast, did not affect the priming phase but became important later in the regeneration process. Deficiencies in GF production led to a linearly increasing delay in liver mass recovery (Fig. 8a). This delay eventually led to a suppression of overall mass recovery. Low GFs mediated this suppression by reducing the fraction of hepatocytes in the replicating phase of the cell cycle (Fig. 8b and c). Unlike inflammatory signaling, however, GF signaling deficiencies did not change the timing of peak regeneration. In order to shift the timing of peak regeneration, it was necessary to lengthen the duration of the cell cycle (Fig. 8d). Decreasing the cell cycle progression rate coupled with a decrease in GF bioavailability not only decreased the magnitude of the cell cycle response, but also delayed the peak response by desynchronizing hepatocyte entry into the cell cycle (Fig. 8e).

### Translating among species using the computational model

We tested whether translating among species can potentially be achieved simply by adjusting model parameters in the extended computational model. Prior to simulation, we sought to identify which parameters likely change among species. The cell cycle duration is known to be fairly consistent across mammalian species; therefore, we maintained this parameter at nominal levels [17–20]. Similarly, the JAK-STAT pathway is understood to be ubiquitous in mammalian species. Therefore, we maintained JAK-STAT signaling pathway parameters constant across species. Additionally, while the physiological parameters used to approximate multiple pathways may indeed change between species, there is little reason to believe that the essential mechanisms of these pathways differ any more than the JAK-STAT signaling pathway does. Therefore we maintained the physiological parameters at nominal levels as well. This assumption of consistent pathway behavior across species does not take into account any differences in network dynamics caused by species-specific molecular dynamics, for example rat IL-6 half-life in rat macrophages compared to human IL-6 half-life in human macrophages.

We considered an approach where all molecular driving events were maintained constant between species, leaving the metabolic demand parameter and the cell growth rate parameter as the only ones available for modification. It has been shown that metabolic demand of an organism is proportional to the mass of the organism raised to an exponential power (estimated to be between 2/3 and 3/4); this is true for both plants and animals and appears to be an organizing principle of biology [21–23]. The metabolic demand parameter is a lumped parameter approximating extrinsic signals that occur in parenchymal and non-parenchymal cells and intrinsic hepatocyte capacity to respond to these signals; however, a portion of these signals may be caused by increased nutrient and toxin flux. Therefore, this term represents, at least in part, a metabolic response to these fluxes, which may vary among species according to overall mass. Lumping extrinsic and intrinsic drivers of regeneration into one parameter makes it difficult to simulate experiments where hepatocytes from one species are transplanted into another, but such a technique is appropriate when considering each species individually [24]. In addition to metabolic demand potentially changing across species, it is possible that cell growth rate may also differ across species. We were able to find no studies reporting grossly observable differences in cell growth rates, while several studies have suggested that the cell growth rate across species appeared to be fairly similar among mammalian species [25, 26]. These results led us to believe that cell growth rate likely changes among mammalian species, but that change is likely not orders of magnitude different. Therefore, we changed the cell growth rate and metabolic demand parameters across species in our model to simulate regeneration in multiple species.

We fit regeneration profiles of rats, mice, and humans by simultaneously changing only the hepatocyte growth rate and metabolic demand parameters and minimizing the sum of squared error between experimental data and simulation output. For rats and mice, the growth rates estimated using this least squares approach were fairly similar (G = 3.5x10^−4^ and 9.7x10^−4^ mass equivalent doublings/min, respectively). The optimum fit for humans, however, resulted in a much higher estimated growth rate (G = 2.5x10^−2^ mass equivalent doublings/min). This estimation is inconsistent with literature suggesting cell growth rate is fairly similar among mammalian species [25, 26]. We therefore constrained human hepatocyte growth rate to the average of rat and mouse growth rates (G = 6.6x10^−4^ mass equivalent doublings/min) (Additional file 13: Table S1) and changed only the metabolic demand parameter to fit human regeneration data.

**Fig. 9:**
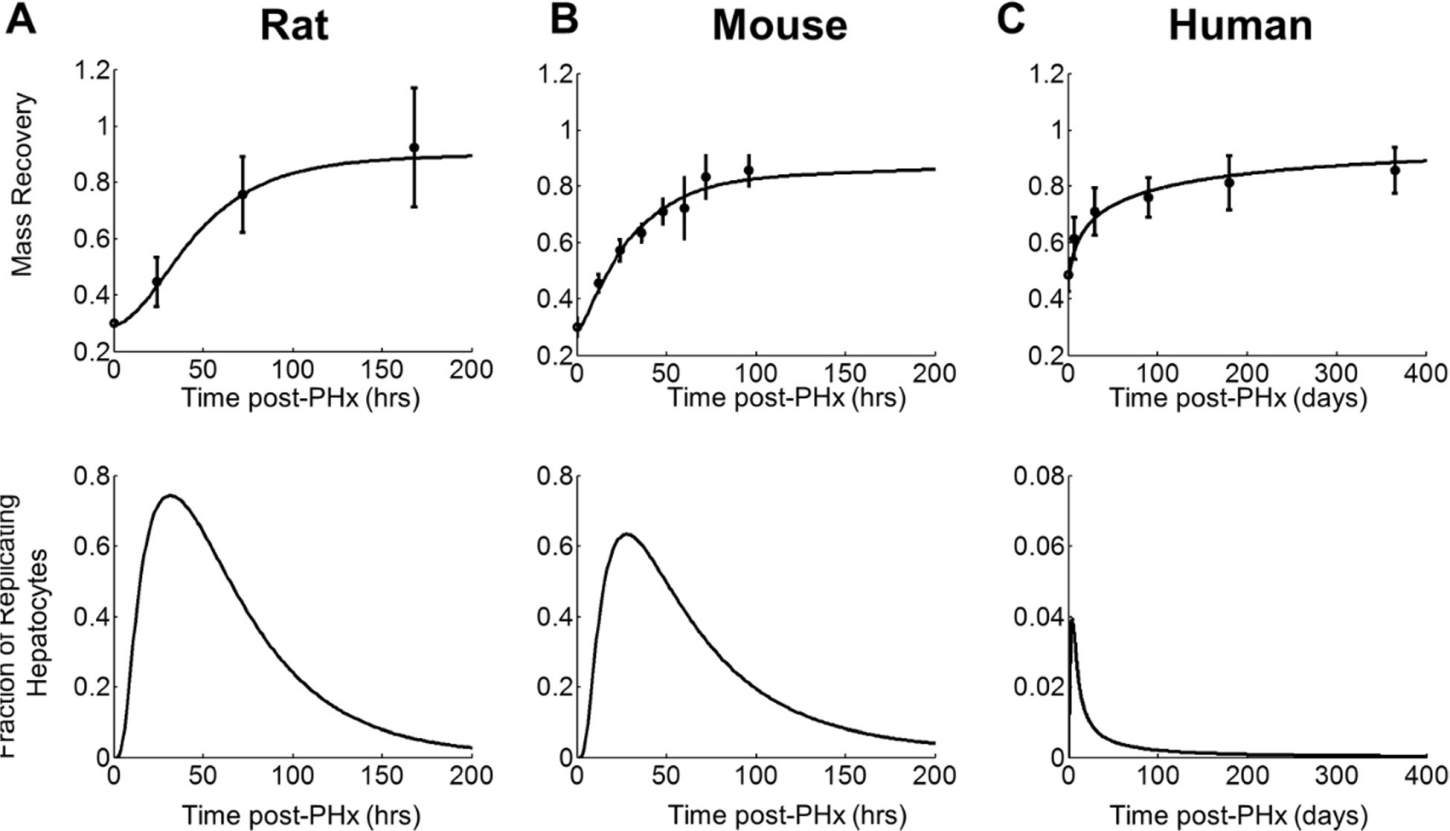
Cross-species comparison of **a** rat, **b** mouse, and **c** human (upper panel) mass recovery profiles with experimental liver regeneration data [16, 27, 28] and (lower Panel) predicted fraction of replicating hepatocytes for each species. This model suggests that the key difference governing regeneration profiles between species is an altered balance between proliferative and replicative balance in hepatocytes. This species-specific balance alters the levels of GFs available during regeneration thereby altering the simulated BrdU incorporation of hepatocytes post-PHx. Rats and mice have similar metabolic loads and growth rates causing similar BrdU incorporation with several slight differences. Rats have a slightly later peak BrdU than mice (30 h vs. 28 h) and a higher peak value (0.75 vs. 0.66). Mouse BrdU incorporation, however, continues longer than rat. Similarly, humans show a reduced peak replication response (note change of scale) but a lengthened regeneration period, leading to similar overall recovery

By modifying only hepatocyte growth rate and metabolic demand parameters, and appropriately scaling the apoptosis parameter θ_ap_, we were able to fit regeneration profiles from rats, mice, and humans post-hepatectomy (Fig. 9a-c) [16, 27, 28]. We scaled the apoptosis parameter θ_ap_ by multiplying θ_ap_ by the ratio of M_mouse/human_ to M_rat_. Both rats and mice regenerate to the initial level of liver mass within ~168 h post-hepatectomy (7 days), while humans take nearly 100 days to recover mass fully. Both rats and mice had a robust response to partial hepatectomy, with an early spike in regenerating cells (peaking near 30 h post-PHx). Rats appeared to have a slightly higher regeneration peak (Fig. 9a), while mice appeared to sustain regeneration slightly longer than rats (Fig. 9b). Although these results did not capture the shift in peak hepatocyte replication from 24 h post-PHx in rats to 48 h post-PHx in mice, fitting the mass recovery dynamics between these two rodent species underscore the similarity in regeneration response between them. Humans, on the other hand, showed a lower peak regeneration but sustained regeneration across many months rather than days (Fig. 9c).

After fitting the metabolic demand parameter to experimental data, we determined an empirical relationship for determining the metabolic demand parameter from the organism body mass, Equation 1 (Additional file 14: Figure S12). We used a power-law expression to describe the relationship between these terms because of the well-known power-law relationship between organism mass and metabolic function.

1 $$ Metabolic\  Demand=47.315\ast Mas{s}^{-0.1825} $$

We have shown that it is possible that the difference in time necessary to regenerate fully is due predominantly to the differential functional demands of the liver across species. Rodents, which live in an environment more prone to infection and liver injury, may require a higher metabolic demand (a component of which is the nutrient delivery per cell) to maintain healthy liver function than humans, which live in a relatively clean environment. Because blood flow and overall nutrient delivery does not change following PHx, a smaller number of cells are receiving a relatively increased nutrient delivery in all species. It is possible that post-PHx the relative increase in metabolic demand per cell—and therefore the driving force for regeneration—may be higher in rodents than in humans.

Liver mass recovery is a much longer process in humans than in rats, lasting months rather than weeks. While our assumptions allowed us to model liver regeneration in humans, other alternative hypotheses about the differences in liver regeneration between rats and humans remain possible. We therefore tested several alternate hypotheses that may be able to explain the differences in regeneration profiles between rats and humans. We tested the hypotheses that humans have an altered stress response compared to rats (Hyp 1); that humans have altered matrix remodeling dynamics and ECM-GF binding compared to rats (Hyp 2); that human hepatocytes have an altered transition time between physiological states (Hyp 3); and that human hepatocytes have a longer cell cycle, a higher apoptosis rate, a higher requiescence rate, and an altered transition rate between physiological states, as was assumed by Periwal et al. [29] (Hyp 4). The study by Periwal et al. [29] reduced the values of parameters controlling the hepatocyte cell cycle rate, apoptosis rate, and requiescence rate by a factor of 24, roughly the difference in lifespan between rats and humans. Additionally, they used clinical data to fit the three physiological parameters governing the rate of hepatocyte transition between states (k_P_, k_R_, and k_Q_), reasoning that since these parameters abstract multiple signaling pathways and regulation, these parameters are most likely to be altered between species. Table 2 contains the parameters used to test these hypotheses. We compared these hypotheses to our hypothesis that an altered metabolic demand can account for differences in liver regeneration dynamics between humans and rats (Hyp 5).

**Table 2:** Summary of parameters used to simulate alternate hypotheses of how human liver regeneration differs from rat liver regeneration

Hyp 1: Altered cytokine response	Hyp 2:Altered GF storage and ECM balance	Hyp 3: Altered state transition rate	Hyp 4: From Periwal et al. [29]	Hyp 5: Reduced metabolic demand
k_IL6_ = 0.1435	κ_deg_ = 4.955	k_QP_ = 1.4x10^−3^	k_QP_ = 1.1x10^−3^	M = 20.8217
κ_IL6_ = 0.4942	κ_ECM_ = 56.30	k_PR_ = 1.5x10^−3^	k_PR_ = 2.6x10^−3^	G = 3.474x10^−4^
V_JAK_ = 1.364x10^3^	k_GF_ = 3.288x10^−3^	k_RQ_ = 70.9x10^−3^	k_RQ_ = 135x10^−3^	
K_m_^JAK^ = 7.565x10^3^	κ_GF_ = 2.139x10^−3^		k_req_ = 4.17x10^−3^	
κ_JAK_ = 0.0398	k_up_ = 0.1008		k_ap_ = 4.17x10^−3^	
[STAT3] = 2.031			k_prol_ = 8.33x10^−3^	
k_STAT3_ = 1.109x10^3^				
K_m_^STAT^ = 0.5178				
κ_SOCS_ = 0.1682				
K_I_^SOCS3^ = 0.0569				
k_IE_ = 18.60				
K_m_^IE^ = 88.13				
κ_IE_ = 1.148				

**Fig. 10:**
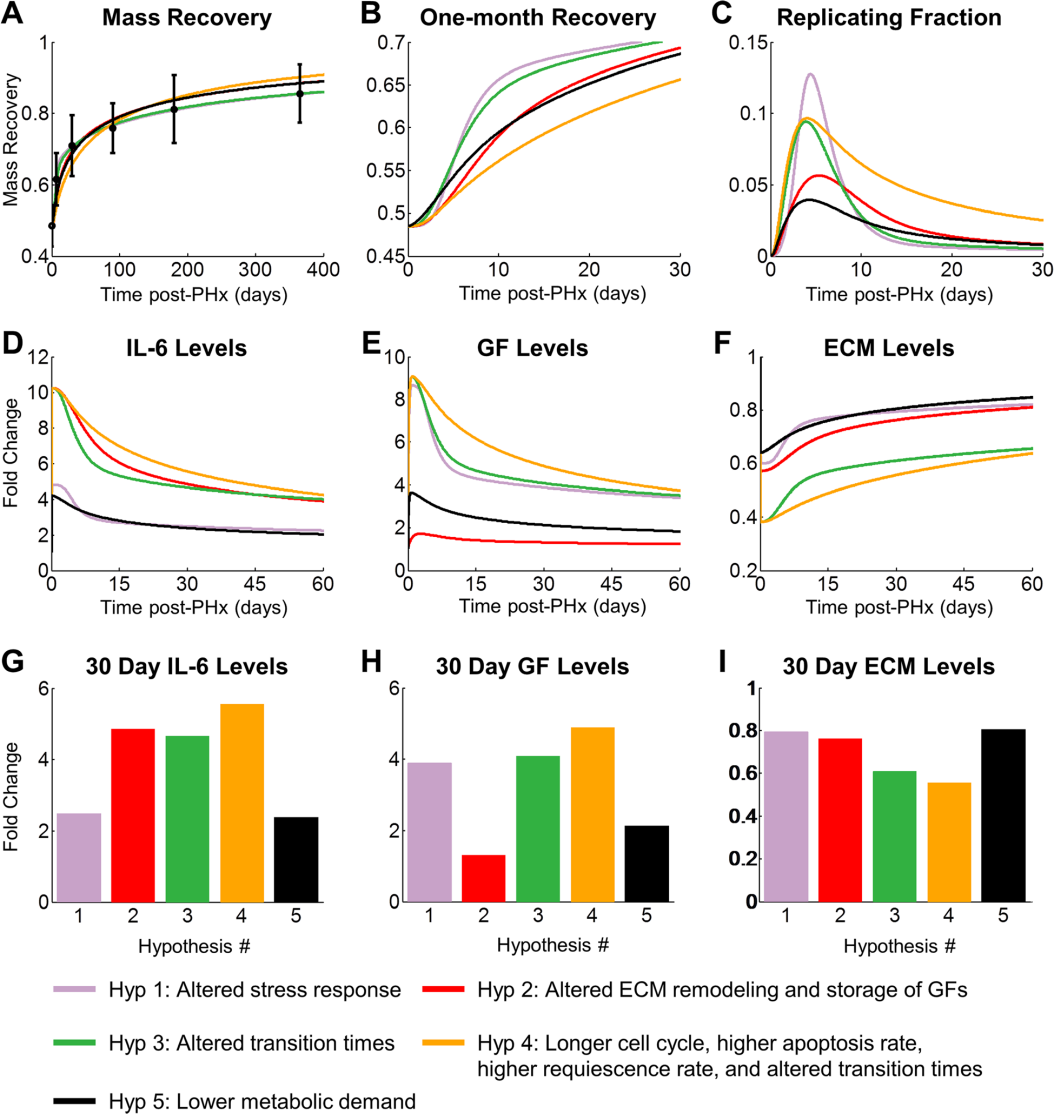
Alternate parameter changes that can reproduce experimental liver regeneration profiles in humans. Parameters were varied to fit experimental data of human mass recovery to test several possible hypotheses about how human liver regeneration differs from rat: the hypothesis that humans have a higher stress response than rats (blue, MSE = 6.1x10-3), the hypothesis that humans store a greater quantity of growth factors in the ECM that is liberated early post-PHx and may have an altered ECM production/degradation balance (red, MSE = 6.25x10-3), the hypothesis that human hepatocytes have a higher transition time between physiological states (green, MSE = 0.25x10-3), the hypothesis that humans have a longer cell cycle, a higher apoptosis rate, a higher requiescence rate, and a higher transition rate between physiological states as was assumed by Periwal et al. [29] (magenta, MSE = 12.16x10-3), and the hypothesis that only the metabolic demand parameter changes (black, MSE = 4.91x10-3). **a** Simulated mass recovery compared to experimental data [28]. **b** Mass recovery over the first 30 days following resection. **c** Fraction of replicating cells (simulated BrdU incorporation) post-resection. **d** IL-6 levels post-resection. **e** GF levels post-resection. **f** ECM accumulation post-resection. It may be possible to differentiate between most of these hypotheses by measuring at 30 days post-resection (**g**) IL-6, (**h**) GF, and (**i**) ECM. To differentiate between the high transition time hypotheses (green) and the hypothesis presented by Periwal et al. (orange), it may also be necessary to measure mass recovery. Approximately two weeks post-resection showed the maximum difference between mass recovery between these two hypotheses. MSE = Mean Squared Error between experimental and simulated data

We found that all of the proposed hypotheses were able to explain human liver regeneration fairly well (Fig. 10a). The early dynamics of regeneration, however, were able to differentiate between many of the hypotheses (Fig. 10b,c). At two weeks post-PHx (14 days), the liver mass recovery should be able to differentiate between several of the hypotheses (Fig. 10b). The biological variability in human liver mass recovery, however, may make this approach challenging. If liver biopsies are available, the fraction of replicating hepatocytes in these samples could be used to identify which (if any) of these hypotheses is correct. Because biopsies of regenerating livers may not be beneficial to regeneration, it may be more clinically feasible to investigate cytokine and growth factor levels in the blood, assuming that they correlate to what is in the liver. Our model predicts that investigating cytokine levels (Fig. 10d and g), growth factor levels (Fig. 10e and h), and ECM accumulation (Fig. 10f and i) at two weeks post-PHx will provide a surrogate for replication fraction to differentiate between hypotheses. The hypotheses that cell transition time differs between species (Hyp 3) and that cell transition time, replication rate, requiescence rate, and apoptosis rate differ between species (Hyp 4) gave similar predictions for molecular regulation at 30 days post-PHx; therefore, to differentiate between these hypotheses, it may be necessary to also investigate mass recovery or replicating fraction of cells. Furthermore, when measuring molecular levels in blood of patients, the fold changes may not match exactly the fold changes predicted to exist in the tissue from model simulations. What should allow for differentiation of hypotheses is the patterns of molecular regulation across multiple proteins.

We varied sets of model parameters to fit simulated regeneration dynamics to experimental human liver regeneration data to predict how human liver regeneration would have to differ from rat liver regeneration for these hypotheses to hold true. If the human cytokine response to PHx is entirely responsible for human to rat differences in regeneration dynamics (Hyp 1), then the production of pro-inflammatory cytokines should be suppressed in humans. Similarly, the hepatocyte response to these inflammatory cytokines should be suppressed as well. This would lead to decreased pro-inflammatory cytokine signaling (Fig. 10g – Hyp 1), causing low expression of MMPs and sustained high levels of ECM (Fig. 10i – Hyp 1). If ECM remodeling and GF signaling is entirely responsible for human to rat differences (Hyp 2), then GF production in humans should be slower than rats and human ECM should be more efficient in binding GF than rat ECM. This would lead to low levels of GF (Fig. 10h – Hyp 2) and high levels of ECM (Fig. 10i – Hyp 2). If the only difference between human and rat regeneration is hepatocyte transition time between physiological states (Hyp 3), then the transitions from Q to P and P to R promoting regeneration should be slower, while the transition from R to Q should be faster in humans than rats. If the assumptions made by Periwal et al. are true (Hyp 4), then the transition times should respond the same way. These longer transition times promoting regeneration lead to similar molecular profiles for these two cases, with high levels of cytokines and growth factors. Therefore, it becomes necessary to measure mass recovery to differentiate between these two hypotheses. The hypothesis that cell show altered transition times (Hyp 3) predicts a higher mass recovery at two weeks than the hypothesis proposed by Periwal et al. (Hyp 4). The difference in mass recovery at two weeks is caused by the assumption of a slower cell cycle, requiescence rate, and apoptosis rate by Periwal. et al. (Fig. 10b – Hyp 4). Our hypothesis that lower metabolic demand is responsible for the differences between human and rat regeneration (Hyp 5) caused a lower overall response to PHx in human than in rats, but one that was sustained over a longer time period. This would lead to suppressed cytokine and GF signaling (Fig. 10g and H – Hyp 5) as well as relatively high levels of ECM (Fig. 10i – Hyp 5), because of low cytokine-induced MMP production. Patterns of molecular regulation that could differentiate hypotheses are summarized in Table 3. We recognize, however, that further experimental results in humans and further model refinement to include absolute molecular quantification and factors not included in the current model may be required to differentiate fully between hypotheses.

**Table 3:** Patterns of molecular regulation (30 days) and mass recovery (14 days) that could differentiate hypotheses of mechanisms underlying liver regeneration in humans

Hypothesis	IL-6 / Inflammation	GF	ECM	Mass Recovery
(1) Altered Inflammation	Moderate	High	High	High
(2) Altered ECM remodeling and GF storage	High	Moderate	High	Moderate
(3) Altered transition times	High	High	Moderate	High
(4) Parameter changes assumed in Periwal et al. [29]	High	High	Moderate	Low
(5) Lower metabolic demand	Moderate	Moderate	High	Moderate

### Predicting effects of chronic disease on liver repair following partial hepatectomy

**Fig. 11:**
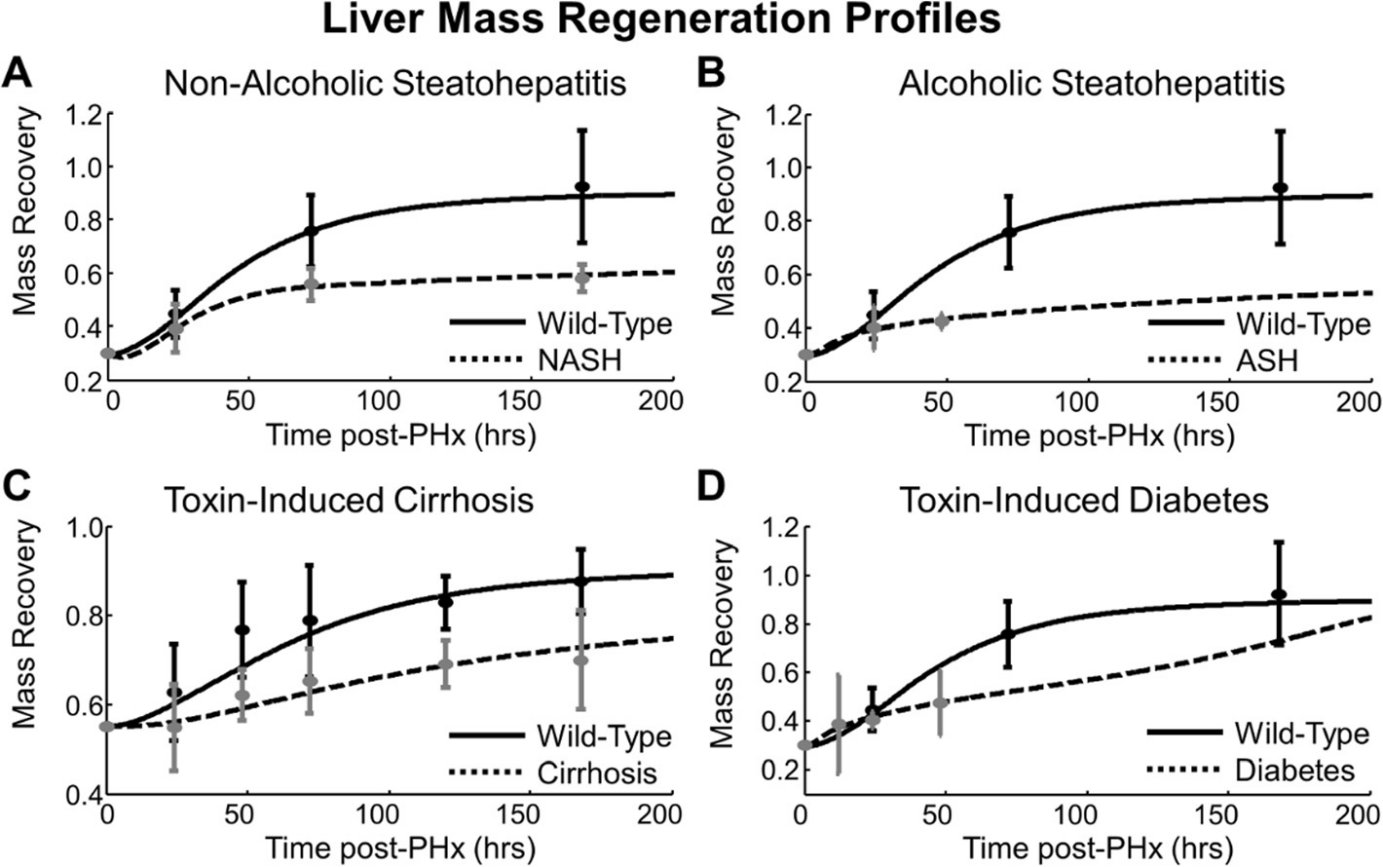
Model fit to disease regeneration profiles revealed altered non-parenchymal cell activity coupled with imbalances in the growth/replication propensity of hepatocytes were sufficient to explain disease-induced inhibition of regeneration. **a** Non-alcoholic steatohepatitis [16], **b** Alcoholic steatohepatitis [41], **c** Cirrhosis [43], and **d** Diabetes [44]

Just as non-alcoholic steatohepatitis (NASH), alcoholic steatohepatitis (ASH), cirrhosis, and diabetes affect liver function differently, each affects liver repair differently as well (Fig. 11). Both non-alcoholic and alcoholic steatohepatitis suppress liver repair following partial hepatectomy as early as 48 h post-surgery and lead to a sustained mass recovery deficit (Fig. 11a,b). Toxin-induced cirrhosis also suppresses regeneration, causing a sustained offset from wild-type regeneration (Fig. 11c). The simulated regeneration profile for diabetic rats suggests that the disease enhances early regeneration but delays full recovery (Fig. 11d). These predictions are consistent with literature reporting that alloxan-induced diabetic rats show a delay in regeneration but no suppression of overall recovery [30]. In humans, studies have shown that diabetes results in a higher risk of post-operative liver failure and death in the first 90 days following liver resection, but when followed for longer than 6 months, diabetes causes no increase in the risk of complication or death, indicating that diabetes may impact the early stages of regeneration greater than the later stages [31, 32].

We tested the hypothesis that alterations to non-parenchymal cell activation are sufficient to explain altered regeneration in these disease phenotypes. We found that despite the differences in repair dynamics, each of these regeneration phenotypes could be modeled by changing a relatively small number of parameters (9 out of 33), including metabolic load, hepatocyte growth rate, and parameters associated with non-parenchymal cells (Additional file 15: Table S2). This result indicates that altered non-parenchymal cell activation is sufficient to explain altered regeneration in these disease phenotypes. We investigated how these parameters change between disease conditions to predict of how diseases could impair regeneration by modulating non-parenchymal cell activation (Additional file 16: Figure S13; Additional file 17: Figure S14; Additional file 18: Figure S15; Additional file 19: Figure S16). A summary of our predictions of disease-impaired regeneration characteristics is available in Table 4. NASH inhibited regeneration mainly through impaired priming (Additional file 16: Figure S13). NASH also caused an inhibited replication response following PHx, which was likely caused by low priming rather than GF deficiencies. Impaired priming and reduced replication caused a majority of mass recovery to occur through cell growth rather than replication. Although its regeneration profile is similar to NASH’s, ASH showed a robust priming response, but it inhibited regeneration mainly through deficiencies in GF bioavailability and ECM remodeling (Additional file 17: Figure S14). The slight increase in liver mass was caused predominantly by cell growth rather than replication. Toxin-induced cirrhosis caused an enhanced priming response in hepatocytes but a reduced replication response (Additional file 18: Figure S15). Reduced GF bioavailability coupled with high levels of ECM reduced the overall regenerative potential of cirrhotic livers. In contrast to NASH and ASH, the mass recovery in cirrhosis was mainly due to cell replication rather than mass increase. Diabetes also inhibited regeneration through deficiencies in GF signaling (Additional file 19: Figure S16). These GF deficiencies caused a delay in the initiation of replication. It is likely that the early enhanced mass recovery in diabetic rats may be due predominantly to hepatomegaly, while eventual mass recovery may be due to replication. Although the exact timing and magnitude of deficiencies in inflammation and GF signaling were not the same for all chronic disease states, all the chronic diseases simulated here showed deficiencies in both signaling pathways. This result suggests that many chronic diseases that affect the liver’s repair ability do so in a combinatorial manner, altering the dynamics of inflammatory response and GF signaling.

**Table 4:** Summary of predicted disease effects on liver regeneration

Disease Model	Mass Recovery	Priming	Replication	IL-6 Signaling	GF	ECM
(1) Non-alcoholic Steatohepatitis	Suppressed	Low	Low	Low	High	High
(2) Alcoholic Steatohepatitis	Suppressed	Sustained	Low	Sustained	Low	High
(3) Toxin-induced Cirrhosis	Suppressed	High	Low	High	Low	High
(4) Allotaxin-induced Diabetes	Delayed	High	Delayed & low	Sustained	Low	Low

Adaptation to chronic diseases also appears to influence the liver’s ability to recover a normal baseline function after an acute challenge. At long times post-PHx, NASH was characterized by sustained high levels of GF signaling, ASH was characterized by sustained high levels of IL-6 and reduced ECM accumulation, and diabetes was characterized by reduced ECM accumulation (Additional file 16: Figure S13 Additional file 17: Figure S14, and Additional file 19: Figure S16). Cirrhosis, on the other hand, was characterized by all molecular levels returning to baseline (Additional file 18: Figure S15). Our prediction of a sustained high inflammatory response in ASH simulations is consistent with previous reports of relatively high levels of inflammatory molecules found in the serum of patients with ASH [33]. This result suggests that one of the fundamental mechanisms of disease progression between ASH and NASH may be a difference in inflammatory response of non-parenchymal cells.

Although our model simulations showed that altered non-parenchymal cell behavior is sufficient to cause impaired regeneration dynamics that are consistent with NASH, ASH, diabetes, and cirrhosis, parenchymal cells likely also contribute to impaired regeneration. We therefore tested whether alterations in hepatocyte response to non-parenchymal cells are sufficient to explain altered regeneration in these same disease phenotypes by changing parameters related to hepatocyte response to non-parenchymal cells (14 out of 33 parameters, Additional file 20: Table S3). We found that for NASH, ASH, and cirrhosis, alterations in hepatocyte response to non-parenchymal cells was also sufficient to explain altered regeneration in these disease phenotypes (Additional file 21: Figure S17A-C). Altering these hepatocyte response parameters was insufficient to explain diabetes-impaired regeneration dynamics (Additional file 21: Figure S17D). In all cases, the previous set of parameters (Additional file 15: Table S2) gave lower mean squared error (MSE) than the hepatocyte-specific parameter alterations (Additional file 20: Table S3). It was interesting to note that the parameter sets used to simulate NASH and ASH eventually resulted in liver failure, with hepatocyte numbers continuing to decrease as the simulation progressed. The results of these simulations, together with the simulations altering non-parenchymal cell behavior and experiments from literature, suggest that disease conditions likely alter the dynamic function of non-parenchymal cells and hepatocytes during liver regeneration. Therefore when investigating liver disease states and response to surgical interventions, a systems-based approach that explicitly accounts for cell-cell interactions is necessary to account for the underlying processes fully.

## Discussion

Our study provides an investigation into the organizational principles and molecular regulation underlying liver regeneration following resection across multiple species and disease states. Our study identified altered modes of regeneration and investigated disease states that cause regeneration to follow these altered modes. This study, however, only addresses surgical resection of the liver and has not been applied to drug-induced liver injury (DILI). Because similar archetypal processes also likely govern liver regeneration following DILI, it is possible that some of the results of our modeling study can be generalized to inform principles underlying regeneration following DILI as well. The altered regeneration dynamics following DILI indicate that additional processes need to be added to the model to accurately capture the complete physiology (for example, clearance of injured or necrotic hepatocytes and immune cell infiltration).

This study investigated liver regeneration through a computational model involving archetypal signaling pathways that represent classes of molecular signaling. Therefore, the simulations in this study suggest relative balances and timing of molecular signals that may be deregulated in disease or altered across species. We have used this approach in a previous study to investigate the molecular factors governing the altered liver regeneration dynamics caused by ablation of the gene adiponectin (Adn). Our modeling approach suggested that the delay and acceleration of regeneration observed in Adn−/− mice was caused by decreased priming in hepatocytes (seen as decreased STAT3 phosphorylation during the first 6 h post-PHx) and enhanced growth factor signaling (observable by 20–40 h post-PHx) [34]. We then measured STAT3 phosphorylation and growth factor levels in liver lysates of Adn−/− mice and found reduced STAT3 phosphorylation at 3 and 6 h post-PHx coupled with high levels of ANG-1, FGF-2, and HGF proteins from 6 to 42 h post-PHx.

Our study suggests several organizational principles of regeneration. Initiation of regeneration appears to be governed by the number of hepatocytes entering the priming phase, which in turn is largely driven by the inflammatory response (modeled as IL-6 signaling). The computational model simulations further suggest that IL-6 signaling activity is amplified at the level of STAT-3 phosphorylation, so that small changes in inflammatory response can cause large changes to STAT-3 phosphorylation and significantly alter the regeneration profile. The timing and magnitude of GF response appears critical to replication, with low or late GF response suppressing overall regeneration. Our results led us to predict that chronic diseases impair liver regeneration through a combination of deficient inflammatory signaling and growth factor bioavailability. We further predicted that these deficiencies are shared between non-parenchymal cell activation and hepatocyte responsiveness to extracellular stimuli.

Our approach allowed us to investigate several hypotheses about how regeneration differs between rats and humans. By maintaining molecular and phenomenological parameters constant across species and modifying metabolic load and hepatocyte growth rate, we were able to fit experimental regeneration profiles across species. This approach has the benefit of conserving hepatocyte-related signaling pathways including the JAK-STAT signaling kinetics across species. These results revealed that regenerative capacity is likely related to animal mass, with larger species having fewer energetic resources to devote to regeneration. This explanation is consistent with identification of peak regeneration in pigs and dogs occurring later than in rats and mice (3 days post-PHx in pigs and dogs, as opposed to 1 day in rodents) [35]. Alternate hypotheses about differences between rat and human liver regeneration dynamics, however, offer different predictions about dynamic tissue behavior post-PHx. We predicted that tissue biopsies and scans taken at two weeks post resection or molecular measurements at one month post resection in humans could differentiate between these hypotheses.

Another factor governing the length of regeneration time is how rapidly hepatocytes are able to increase their functional mass to compensate for lost tissue. Large mass may not be beneficial to liver repair if much of the extra mass does not contribute to liver function; therefore, the mass regained in this simulation can be seen as functional mass increase that contributes to liver function. As opposed to the metabolic demand parameter, hepatocyte growth rate was not related to animal mass. Growth rate may therefore be governed by other factors, such as maximum glucose metabolic flux possible, mitochondrial activity and number of mitochondria, and the relative amount of nutrients available post PHx. By incorporating cell growth, the model proposed in this work was able to capture the rapid increase in tissue mass humans are capable of, up to 70 % of liver mass restored by 30 days after 70 % PHx [28]. Experiments measuring growth rates of hepatocytes in vitro or further hepatectomy experiments performed using pigs or other species can be used to test and refine the simple relationship proposed between metabolic demand and body mass.

According to our analysis, the number of parameters that need to be changed to translate across species is relatively small (a minimum of two). Futhermore, the minimum set of parameters changed were physiological parameters, M and G. This does not mean that there are no differences in molecular regulation across species; it does, however, suggest that the differences are the result of similar processes across species responding to species-specific physiology. This results in altered molecular and regeneration dynamics across species. In contrast, we changed multiple parameters, including parameters related to molecular signaling, to simulate disease effect on liver regeneration. Taken together, these results suggest that biological processes behaving normally can account for differences across species but cannot account for disease effects on regeneration phenotypes.

Although the model describes fairly well experimental data, the model description of the cell cycle does not contain specific phases of the cell cycle. The rate of cell proliferation in the model contains all the steps from exit from the G0 phase to a complete cell division. Therefore, this rate also includes any additional time taken for a quiescent hepatocyte to dedifferentiate, divide, and redifferentiate. Little is known about how long any dedifferentiation and redifferentiation takes or if the time needed for these processes varies across species. Therefore, the overall rate of cell proliferation may vary between species. Although we did not explicitly address this possibility in the current study, further studies could explore this as a potential contribution to the difference in peak hepatocyte replication times between rats and mice.

Parametric sensitivity analysis of the computational model revealed that regeneration is dynamically controlled and that not all factors respond the same across all times. This result coupled with the pulsatile sensitivity analysis recently performed on the original model proposed by Furchtgott et al. [29] indicates that treatments designed to improve regenerative ability during chronic disease or following liver transplant may need to be dynamic as well [34]. Extending the results of simulations of chronic disease states in rats to the human model may assist in scheduling treatments for patients suffering from chronic diseases post-transplantation to maximize regeneration. For example, during the first week (the apparent priming phase in humans) it might be necessary to renormalize hepatocyte response to inflammation signals while later treatments (replication phase) may need to increase growth factor levels.

Our model-based approach offers unique insights into the mechanisms of liver disease progression in the context of chronic disease; however, there are several limitations inherent to this approach. The first limitation is that only the JAK-STAT signaling pathway is explicitly considered in this model. Although this pathway has been shown to be critical for a normal repair phenotype, even a hepatocyte-specific STAT-3 knockout does not completely inhibit regeneration [36]. In this genotype, signaling through ERK compensates for the lack of STAT-3. The importance of the liver’s repair mechanism ensures that multiple compensatory signaling pathways are available to act [11]. Our model can be extended to include additional signaling pathways to account for compensatory signaling and cross-talk. We note, however, that the present simplification involving cell phenotype transitions sufficiently captures major features of the liver regeneration process. Such simplified models have led to important insights into biological regulation in other contexts as well [13, 37, 38].

Another limitation is that the current model takes into account only linear responses of non-parenchymal cells during liver repair. Many reviews highlight the important role of timing of non-parenchymal cell signaling during liver repair [10, 11]. For instance, the critical contribution of non-parenchymal cells has been demonstrated using animals where Kupffer cells have been depleted, thereby significantly delaying regeneration following hepatectomy [14]. The current simulations suggest that Kupffer cells are largely responsible for priming hepatocytes. Hepatic stellate cells appear to be the main regulator of hepatocyte regeneration, governing both proliferation through control of growth factor bioavailability and termination of regeneration through ECM production and degradation. Therefore, moving towards a more comprehensive computational model of liver repair in health and chronic disease requires inclusion of alternative regulatory mechanisms within hepatocytes, as well as the activation and signaling of non-parenchymal cells. To facilitate this integration, one could consider the existing models of macrophage or Kupffer cell activation and hepatic stellate cell activation. For instance, macrophage activation has been studied using a computational model of the cytokine-mediated pathways [39, 40]. Specific to the liver, our group has recently developed a computational model of cytokine-mediated hepatic stellate cell activation that incorporates multiple pathways with cross-talk as well as microRNA mediated regulation [39, 40].

## Conclusions

Our computational model was able to match liver regeneration profiles across multiple chronic disease models and across species. This modeling framework can act as a tool to translate results from rodent experiments to clinically actionable hypotheses in primates or humans. Our study suggests that liver regeneration is dynamically controlled by factors produced by non-parenchymal cells. Inflammatory signaling (predominantly from Kupffer cells) governs the priming response of hepatocytes, while growth factors (predominantly produced by hepatic stellate cells) govern hepatocyte entry into the cell cycle. The synchronicity of hepatocyte entry into the cell cycle is governed by both growth factor levels and timing as well as proliferation rate of hepatocytes. These findings underscore the importance of non-parenchymal cells to recovering the liver’s repair ability from a diseased state. Therefore, future computational work should explicitly take contributions from non-parenchymal cells into account.

## Methods

### Computational model

We used an extended computational model of liver regeneration (represented schematically in Fig. 1) to investigate quantitatively how altering the molecular regulation of hepatocytes affects the liver’s innate repair ability. A detailed explanation of initial model derivation and parameter estimation is available in [13]. Our extended model maintains the framework of the previously published initial model by allowing hepatocytes to exist in one of three states: Quiescent (Q), Primed (P), or Replicating (R). Factors produced by non-parenchymal cells in response to liver metabolic load (metabolic demand per cell or M/N) shift hepatocytes between states, according to the following equations.

2 $$ \frac{d}{dt}Q = -{k}_{QP}\left(\left[ IE\right]-\left[I{E}_0\right]\right)Q+{k}_{RQ}\left[ECM\right]R+{k}_{req}{\sigma}_{req}P-{k}_{ap}{\sigma}_{ap}Q $$

3 $$ \frac{d}{dt}P = {k}_{QP}\left(\left[ IE\right]-\left[I{E}_0\right]\right)Q - {k}_{PR}\left(\left[GF\right]-\left[G{F}_0\right]\right)P - {k}_{req}{\sigma}_{req}P-{k}_{ap}{\sigma}_{ap}Q $$

4 $$ \frac{d}{dt}R = {k}_{PR}\left(\left[GF\right]-\left[G{F}_0\right]\right)P-{k}_{RQ}\left[ECM\right]R+{k}_{prol}R - {k}_{ap}{\sigma}_{ap}R $$

Where [IE] represents the concentration of immediate early genes expressed in response to STAT-3 transcriptional regulation and [ECM] represents the amount of extracellular matrix. σ_ap_ and σ_req_ are sigmoidal functions defined as:

5 $$ {\sigma}_{ap}=0.5\ast \left(1+ \tanh \left(\frac{\left({\theta}_{ap} - \raisebox{1ex}{$M$}\!\left/ \!\raisebox{-1ex}{$N$}\right.\right)}{\upbeta_{\mathrm{ap}}}\right)\right) $$

6 $$ {\sigma}_{req}=0.5\ast \left(1+ \tanh \left(\frac{\left({\theta}_{req}-\left[GF\right]\right)}{\upbeta_{\mathrm{req}}}\right)\right) $$

The parameters β and θ in each of these equations are tuned so that when metabolic load is high, σ_ap_ is high; conversely, when [GF] is high, σ_req_ is low. Therefore, when cells are highly stressed (high metabolic load), apoptosis occurs at a high rate; when GFs are available, cells remain in the “Replicating” state.

The JAK-STAT signaling pathway, GF production, and ECM production are modeled as a combination of first order and Michealis-Menton kinetics, as shown in the following equations. For a schematic of the JAK-STAT signaling pathway, see Fig. 3 a.

7 $$ \frac{d}{dt}\left[IL6\right]={k}_{IL6}\frac{M}{N}-\frac{V_{JAK}\left[IL6\right]}{\left[IL6\right]+{k}_M^{JAK}}-{\kappa}_{\mathrm{IL}6}\left[IL6\right]+{k}_1 $$

8 $$ \frac{d}{dt}\left[JAK\right]=\frac{V_{JAK}\left[IL6\right]}{\left[IL6\right]+{k}_M^{JAK}}-{\kappa}_{JAK}\left[JAK\right]+{k}_2 $$

9 $$ \begin{array}{l}\frac{d}{dt}\left[ STAT3\right]=\frac{V_{ST3}\left[JAK\right]{\left[ proSTAT3\right]}^2}{{\left[ proSTAT3\right]}^2+{k}_M^{ST3}\left(1+\left[ SOCS3\right]/{k}_I^{SOCS3}\right)}\\ {}\kern4.08em -\frac{V_{IE}\left[ STAT3\right]}{\left[ STAT3\right]+{k}_M^{IE}}-\frac{V_{SOCS3}\left[ STAT3\right]}{\left[ STAT3\right]+{k}_M^{SOCS3}}-{\kappa}_{ST3}\left[ STAT3\right]+{k}_3\kern0.6em \end{array} $$

10 $$ \frac{d}{dt}\left[ SOCS3\right] = \frac{V_{SOCS3}\left[ STAT3\right]}{\left[ STAT3\right]+{k}_M^{SOCS3}}-{\kappa}_{SOCS3}\left[ SOCS3\right]+{k}_4 $$

11 $$ \frac{d}{dt}\left[ IE\right]=\frac{V_{IE}\left[ STAT3\right]}{\left[ STAT3\right]+{k}_M^{IE}}-{\kappa}_{IE}\left[ IE\right]+{k}_5 $$

12 $$ \frac{d}{dt}\left[GF\right]={k}_{GF}\frac{M}{N}-{k}_{up}\left[GF\right]\left[ECM\right]-{\kappa}_{GF}\left[GF\right]+{k}_7 $$

13 $$ \frac{d}{dt}\left[ECM\right] = -{k}_{deg\ }\left[IL6\right]\left[ECM\right]-{\kappa}_{ECM}\left[ECM\right]+{k}_6 $$

Where [proSTAT3] represents the concentration of monomeric STAT-3 available to dimerize following IL-6 signaling. It should be noted that in the original model our [IL-6] term representing cytokine signaling was called [TNF]. Cannonically, TNF signals through the NF-κB cascade, while IL-6 signals through the JAK-STAT cascade. Table 1 states that the approximate biological correlate of the [IL-6] variable in the model is the “Cytokine microenvironment of the liver”. As previously described in [13] and in [34], the [IL-6] variable should be considered a lumped variable representing the physiological impact of general cytokine signaling rather than an exact analogue to IL-6 protein levels. Therefore, we used the name [IL-6] for this variable with parameters derived from TNF.

The overall cell mass, N, was modified from the initial model to include cell growth of primed and replicating cells in response to metabolic load as follows:

14 $$ N=Q+G\left(P+R\right) $$

Where G represents the relative cell mass, which is initially set to 1.

Additionally, when considering the contribution of matrix bound factors to the priming phase of regeneration (Fig. 1b, gray portion), the following equations were added.

15 $$ \frac{dMB{F}_{ECM}}{dt} = -{k}_{MBF}\left(\frac{1}{\left[ECM\right]} - {\left[ECM\right]}_0\right) + {k}_{up}\left[GF\right]\left[ECM\right] $$

Where MBF_ECM_ represents the matrix-bound signals, which are released from the matrix when matrix is degraded at a rate of k_MBF_. We assumed that these signaling factors, which could contain growth factors such as HGF and FGF, were replenished at a rate equivalent to growth factor uptake by the ECM.

16 $$ \frac{dMB{F}_{Free}}{dt}={k}_{MBF}\left(\frac{1}{\left[ECM\right]} - {\left[ECM\right]}_0\right) - {\kappa}_{MBF}\left[MB{F}_{Free}\right] $$

Where MBF_Free_ represents the signaling factors released from matrix and κ_MBF_ is the degradation rate of MBF_Free_ once they have been released.

These additional signals (specifically, MBF_Free_) act to prime hepatocytes. We assumed that the transition rate from quiescent to primed was similar no matter whether MBF or IE gene signals were driving the transition. We therefore modified equations 2 and 3 as follows to take this additional signaling into account.

2a $$ \frac{d}{dt}Q = -{k}_{QP}\left(\left[ IE\right]-\left[I{E}_0\right]\right)Q-{k}_{QP}\left[{S}_{Free}\right]Q+{k}_{RQ}\left[ECM\right]R+{k}_{req}{\sigma}_{req}P-{k}_{ap}{\sigma}_{ap}Q $$

3a $$ \frac{d}{dt}P = {k}_{QP}\left(\left[ IE\right]-\left[I{E}_0\right]\right)Q+{k}_{QP}\left[{S}_{Free}\right]Q - {k}_{PR}\left(\left[GF\right]-\left[G{F}_0\right]\right)P - {k}_{req}{\sigma}_{req}P-{k}_{ap}{\sigma}_{ap}Q $$

All simulations were performed in Matlab (Mathworks, Natick, MA). Model equations were set up to prevent molecular levels from becoming negative; however, some parameter sets combined with the integration tolerances of ode15s led to GF levels becoming negative at longer simulation times (greater than 150 h). These impossible GF levels did not significantly impact the regeneration profile because most of the growth had concluded by the time GF became negative. Because of these numerical instabilities, however, GF levels were constrained to a minimum of 1.

### Transforming published data on liver regeneration into fractional recovery of tissue mass

#### High fructose-induced steatosis (NASH) and Controls

In the study by Tanoue et al. [16], male Sprague–Dawley rats (8 weeks old) were fed either a high fructose diet (total calories from 66 % fructose, 11 % fat, and 19 % protein) or a control diet (chow with total calories from 10 % fructose, 12 % fat, and 19 % protein) for a period of four weeks. Rats with high fructose-induced non-alcoholic steatohepatitis (NASH) showed high serum triglycerides, accumulation of hepatic fat, and more severe insulin resistance, indicating a disease state similar to human NASH. Following four weeks of their respective diets, rats were anesthetized with ether and a 70 % partial hepatectomy was performed. Following resection, rats were fed a standard CE-2 diet. During recovery from resection, rats were sacrificed and their regenerating livers were removed and weighed. The data reported in this study were given in “liver regeneration rate”, which is the percentage of liver mass recovered as normalized to the initial remnant liver mass immediately following hepatectomy, according to equation 17 [16].

17 $$ Liver\  Regeneration\  Rate = 100\%\left\{\frac{Final\  Weight-\left( Original\  Weight- Excised\  Weight\right)}{Original\  Weight}\right\} $$

Liver regeneration rate is the fractional mass recovery minus the remnant liver fractions; therefore, we added 30 % to the reported liver regeneration rate to convert liver regeneration rate to fractional mass recovery.

#### Ethanol-induced steatosis (ASH)

In the study by Yang et al. [41], Sprague–Dawley rats (125 g body weight) were fed either a liquid ethanol diet (355 kcal ethanol, 115 kcal carbohydrates, 360 kcal fat, and 180 kcal protein per liter) or a control diet (470 kcal carbohydrates, 360 kcal fat, and 180 kcal protein per liter) for a period of five weeks. After five week adaptation to these diets, rats were anesthetized using ether and a 70 % PHx was performed. Rats were sacrificed at 24 h and 48 h post-PHx, and liver weight was measured. The data presented by Yang et al. [41] were given in percentage of initial weight at 24 and 48 h post-hepatectomy [41]. We assumed that the initial % of initial liver weight was 30 % because a 70 % PHx was performed. Therefore, to convert from % initial liver weight to fractional recovery, we divided % initial liver weight by 100 %. Although we imposed no further constraints on regeneration in rats with ASH, based on observations of ^3^H-thymadine incorporation from previous studies, we surmise that it is unlikely that significant hepatocyte replication occurs beyond 48 h post-hepatectomy in alcohol-fed rats [42].

#### Toxin-induced cirrhosis

In the study by Kaibori et al. [43], 6 week old male Sprague Dawley rats (150-200 g body weight) were injected with thioacetamide (4 % thioacetamide at 20 mg/100 g body weight) thrice weekly for 10 weeks. The rats were then kept for an additional 3 weeks to allow for thioacetamide washout. Cirrhosis was then confirmed by histology. Following development of cirrhosis, rats were anesthetized with ether and 45 % partial hepatectomy was performed. Rats were sacrificed and their livers were excised and weighed at 1, 2, 3, 5, and 7 days post-PHx [43].

At the time of PHx, remnant cirrhotic livers from 10 additional rats were weighed as a measure of original remnant liver weight. Liver regeneration rate was calculated as follows:

18 $$ Liver\  Regeneration\  Rate=\left(\frac{Remnant\  weight}{Original\  weight}\right)\ast 100\% $$

Therefore, the only conversion necessary to convert liver regeneration rate to fractional mass recovery was to divide by 100 %.

#### Toxin-induced type 1 diabetes

In the study by Johnston et al. [44], diabetes was induced in male Wistar rats (200-300 g body weight) by administering a single dose of streptozotocin (65 mg/kg body weight) injected into the tail vein under light anesthesia (ether). Rats then received 0.28 M glucose to drink. Partial hepatectomy was performed five days following streptozotocin administration. During recovery, rats were sacrificed and dry liver weight was measured at 12, 24, and 48 h post-resection. The data reported in this study were given in liver dry weight percent of total body weight.

To convert these data to fractional recovery, we first calculated the baseline liver dry weight to total body weight percent. From the 10 week-old organ weights of the Phenome project at the National BioResource Project for the Rat in Japan (www.anim.med.kyoto-u.ac.jp/nbr), we found that the average liver to body weight percent across rat strains (and specifically for the WST.F334-*Kmch/*Kyo strain) is approximately 3 %. Johnston et al. [44] states that water content in the livers of sham-operated rats was 64.9 %. Thus, the following two equations were constructed to solve for initial dry liver to body weight percent.

19 $$ \frac{\left( Dry\  Liver\right)+(Water)}{Body\  Weight}=3\% $$

20 $$ \raisebox{1ex}{$ Dry\  Liver$}\!\left/ \!\raisebox{-1ex}{$25.1\%$}\right. = \raisebox{1ex}{$ Water$}\!\left/ \!\raisebox{-1ex}{$64.9\%\ $}\right. $$

where *Dry Liver* and *Water* are the weights of the dry tissue and water content of the tissue. Equation 20, can be rearranged as follows.

21 $$ Water=2.586(Liver) $$

Thus, equation 19 can be solved for baseline dry liver to body weight percentage by inserting equation 21 into equation 19 to yield baseline dry liver to body weight percentage was 1.16 % in the rats used in this study. A 70 % PHx yields a starting dry liver to body weight percentage of 0.348 % corresponding to a fractional recovery of 0.3. All data in this study were therefore scaled by a factor of 0.3/0.348 % to convert the dry liver to body weight percentage to fractional recovery [44].

Previous studies have suggested that alloxan-induced diabetic rats showed a delay in regeneration but that diabetes did not suppress overall recovery [30]. We therefore constrained recovery at 300 h post-PHx in diabetic rats to be the same as for wild-type rats.

#### Mouse liver regeneration

Male mice aged 8–12 weeks (129S1) were fed standard mouse chow *ad libitum*. Mice were anesthetized by pentobarbital and 70 % PHx was performed. The data from Shu et al. [27] for control mice were given in liver to body weight ratio. To convert these data to fractional recovery, these data were scaled by 0.3 divided by initial value for liver-to-body weight ratio.

#### Human liver regeneration

The data presented by Periwal et al. [29] were already given as the fraction of original liver volume, hence requiring no conversion. Similarly, the data presented by Pomfret et al. [28] were given in percent regeneration, which is defined as remnant volume divided by original volume (x100 %). No conversion was required for these data as well.

### Sensitivity analysis

Normalized sensitivity coefficients were estimated by changing each parameter (p_i_) by +/− 10 % of its nominal value and calculating sensitivity at each simulation time point according to equation 22.

22 $$ {S}_{\mathrm{i}}(t)=\frac{\Delta Mass(t)/ Mass(t)}{\Delta {p}_i/{p}_i} $$

*Mass(t)* represents the nominal mass fraction of hepatocytes at any given time, *t*, and *ΔMass(t)* is the deviation from nominal caused by the parameter change. The result is a dynamic parametric sensitivity, showing how the profile of liver regeneration responds to changes in parameters as a function of time.

### Statistical methods

We performed a log-likelihood ratio test to assess whether our extended model described the experimental data significantly better than the previous model. This test takes into account the number of parameters used in the model and the model error in fitting the experimental data. We assumed that the residuals from the fitted models followed a Gaussian distribution (i.e. there was no non-random pattern to the residuals) and used one degree of freedom, corresponding to the cell growth parameter we added to the model. In the case of this model comparison, the original model has 1 fewer parameters than the extended model, we therefore considered the extended model as the unrestricted model. For each model, the log-likelihood function was used to calculate the model fit to experimental data from Tanoue et al. [16] in accordance with equation 23.

23 $$ l\left(\mu, {\sigma}^2;{x}_1,{x}_2, \dots,\ {x}_n\right) = -\frac{n}{2} \ln \left(2\pi \right)-\frac{n}{2} \ln \left({\sigma}^2\right)-\frac{1}{2{\sigma}^2}\ {\displaystyle {\sum}_{j=1}^n{\left({x}_j-\mu \right)}^2} $$

Where μ and σ^2^ were estimated from the residuals for each model.

The ltestratio function in Matlab was used to compare the likelihood of the two models.

### Availability of supporting data

No datasets were generated in this study. The model used in this study is available as a supplemental file.

## Additional files

Additional file 1: Figure S1. Simulated metabolic load (M/N) during liver regeneration following 70 % PHx in rats for conditions including cell growth and without cell growth. (TIFF 60 kb) Additional file 2:
**Matlab code executing the model used in this study.** (M 10 kb) Additional file 3: Figure S2. Relative levels of IE genes (Fold Change) and signals released from the ECM (Relative Amount) during the priming phase. Model parameters were scaled in such a way that priming signals from Kupffer cells and from initially matrix-bound factors had relatively equal contributions to hepatocyte priming. The peak of MBF signaling occurred at approximately 45 min post-PHx. (TIFF 76 kb) Additional file 4: Figure S3. Comparison of simulated mass recovery with and without considering Matrix Bound Factors (MBF). (A) Including EBF signaling in the computational model did not significantly change the overall dynamic mass recovery profile. (B) Including EBF signaling impacted the onset timing of regeneration leading to a small offset between dynamic regeneration including and excluding EBF signaling. This small offset (~0.006) remained throughout regeneration. (TIFF 1021 kb) Additional file 5: Figure S4. Model-predicted molecular regulation profiles underlying delayed regeneration response. (A) Mass recovery of specific delayed cases of liver regeneration compared to nominal and (B) molecular regulation for each regeneration profile. Deficiencies in either priming signals or growth factor bioavailability can lead to delayed regeneration. Note that parameters related to hepatocyte response to these signals change in addition to parameters governing molecular regulation. Dashed line represents nominal profile, black line represents the profile corresponding to delayed regeneration. (TIFF 429 kb) Additional file 6: Figure S5. Model-predicted molecular regulation profiles underlying suppressed regeneration response. (A) Mass recovery of specific suppressed cases of liver regeneration compared to nominal and (B) molecular regulation for each regeneration profile. Suppressed regeneration can be caused by deficient priming signals or deficient growth factor bioavailability. Dashed line represents nominal profile, black line represents the profile corresponding to suppressed regeneration. (TIFF 426 kb) Additional file 7: Figure S6. Model-predicted molecular regulation profiles underlying enhanced regeneration response. (A) Mass recovery of specific enhanced cases of liver regeneration compared to nominal and (B) molecular regulation for each regeneration profile. Enhanced regeneration can be regulated by enhanced GF bioavalability or by enhanced hepatocyte response to the presence of growth factors. Dashed line represents nominal profile, black line represents the profile corresponding to enhanced regeneration. (TIFF 449 kb) Additional file 8: Figure S7. Model-predicted molecular regulation profiles underlying delayed and enhanced regeneration response. (A) Mass recovery of specific delayed and enhanced cases of liver regeneration compared to nominal and (B) molecular regulation for each regeneration profile. One model-predicted cause of this profile is deficient priming signals coupled with enhanced growth factor bioavailability. Dashed line represents nominal profile, black line represents the profile corresponding to delayed and enhanced regeneration. (TIFF 437 kb) Additional file 9: Figure S8. Model-predicted molecular regulation profiles underlying unresponsive regeneration response. (A) Mass recovery of specific unresponsive cases of liver regeneration compared to nominal and (B) molecular regulation for each regeneration profile. Both regeneration profiles and molecular regulation appear to be unresponsive in the profiles investigated. Dashed line represents nominal profile, black line represents the profile corresponding to unresponsive regeneration. (TIFF 392 kb) Additional file 10: Figure S9. Model-predicted molecular regulation profiles underlying liver failure. (A) Mass recovery of specific liver cases of liver failure compared to nominal regeneration and (B) molecular regulation for each liver failure profile. As the liver fails, Kupffer cells and hepatic stellate cells attempt to rescue the liver by producing more pro-regenerative factors. Dashed line represents nominal profile, black line represents the profile corresponding to liver failure. (TIFF 420 kb) Additional file 11: Figure S10. Potential antagonism between pairs of parameters. We used a local sensitivity analysis to identify pairs of parameters that impact the levels of a single factor or are closely related but have opposing effects on overall mass recovery. In addition to metabolic demand and growth rate (Fig. 5c) this analysis identified (A) IL-6 production and degradation and (B) GF production and degradation as potentially antagonistic parameter pairs. (TIFF 965 kb) Additional file 12: Figure S11. Heatmap comparison of overall liver mass recovered for altered GF production and degradation rates. (A) Heatmap with rescaled color mapping showing a subtle effect of coarse-grained control of regeneration by GF production and fine-tuned control by GF degradation (legend below), (B) Mass recovery holding one parameter constant at 20 and varying the other, and (C) Mass recovery holding one parameter constant at 20 and varying the other between 0–20. All parameter changes are displayed in Fold change [FC] over nominal parameter value. The black line indicates when GF production rate fold change equals GF degradation rate fold change. Asymmetry around this line, as in (A), indicates a differential effect of GF production and degradation. (TIFF 235 kb) Additional file 13: Table S1. Parameter changes to simulate regeneration in multiple species (DOCX 11 kb) Additional file 14: Figure S12. Relationships between fitted parameters and body mass across species. (A) Metabolic demand shows a negative exponential relationship with body mass following the equation: *Metabolic Demand* = 47.315 ∗ *Mass* ^− 0.1825^ (R^2^ = 0.95). (B) Cell growth rate for humans was estimated as the average growth for mouse and rat because there is little difference in cultured hepatocyte growth rates between species. (TIFF 66 kb) Additional file 15: Table S2. Parameter changes to simulate alternate regeneration conditions. (DOCX 13 kb) Additional file 16: Figure S13. Regeneration profiles for healthy livers and those with high fat, fructose-induced steatosis. (A) NASH causes an inhibited replication response following PHx. The majority of mass recovery is caused by cell growth rather than replication. (B) This regeneration profile is driven by a lack of inflammatory signaling, leading to reduced priming. Although GFs are available, the low priming means that few hepatocytes are available to enter the replication stage. (MSE = 2.25x10^−4^). (TIFF 302 kb) Additional file 17: Figure S14. Regeneration profiles for healthy livers and those with alcohol-induced steatosis. (A) Alcoholic steatosis causes suppressed liver regeneration, with little mass recovery. The slight increase in liver mass is caused predominantly by cell growth rather than replication. (B) This profile is driven by sustained inflammatory signaling, lack of growth factor bioavailability, and increased matrix deposition following wounding. (MSE = 8.82x10^−5^) (TIFF 294 kb) Additional file 18: Figure S15. Regeneration profiles for healthy livers and those with toxin-induced cirrhosis. (A) Fibrosis causes a delay in the initiation of regeneration (note change in time scale) but ultimately little change in overall mass recovery. Mass recovery is due mainly to hepatocyte replication rather than cell growth. (B) This profile is driven by a sustained inflammatory response, a lack of growth factor bioavailability, and impaired matrix deposition following wounding. (MSE = 2.50x10^−3^) (TIFF 302 kb) Additional file 19: Figure S16. Regeneration profiles for healthy livers and those with diabetes. (A) Diabetes causes enhanced priming but delayed proliferation and delayed mass recovery. (B) This profile is driven predominantly by sustained cytokine signaling and a lack of growth factor bioavailability. (MSE = 4.05x10^−4^) (TIFF 307 kb) Additional file 20: Table S3. Hepatocyte-specific parameter changes to simulate alternate regeneration conditions. (DOCX 12 kb) Additional file 21: Figure S17. Simulations using hepaotyce-specific parameter alterations compared to disease regeneration profiles. Model fits to disease regeneration profiles reveals altered hepatocyte response to non-parenchymal cell signaling is sufficient to explain disease-induced inhibition of regeneration in (A) Non-alcoholic steatohepatitis (MSE = 1.96x10^−2^), (B) Alcoholic steatohepatitis (MSE = 1.89x10^−2^), and (C) Chirrhosis (MSE = 5.14x10^−2^), but not in (D) Diabetes (MSE = 1.19). In all cases, the previous set of parameters (Additional file 15: Table S2) gave lower MSE than the hepatocyte-specific parameter alterations (Additional file 20: Table S3). MSE = Mean Squared Error between experimental and simulated data. (TIFF 1791 kb)
