# Supplementary figures and images for: Systems analysis of non-parenchymal cell modulation of liver repair across multiple regeneration modes
Source: BMC Syst Biol. 2015 Oct 22;9:71. doi: 10.1186/s12918-015-0220-9 (PMC4618752; doi:10.1186/s12918-015-0220-9)

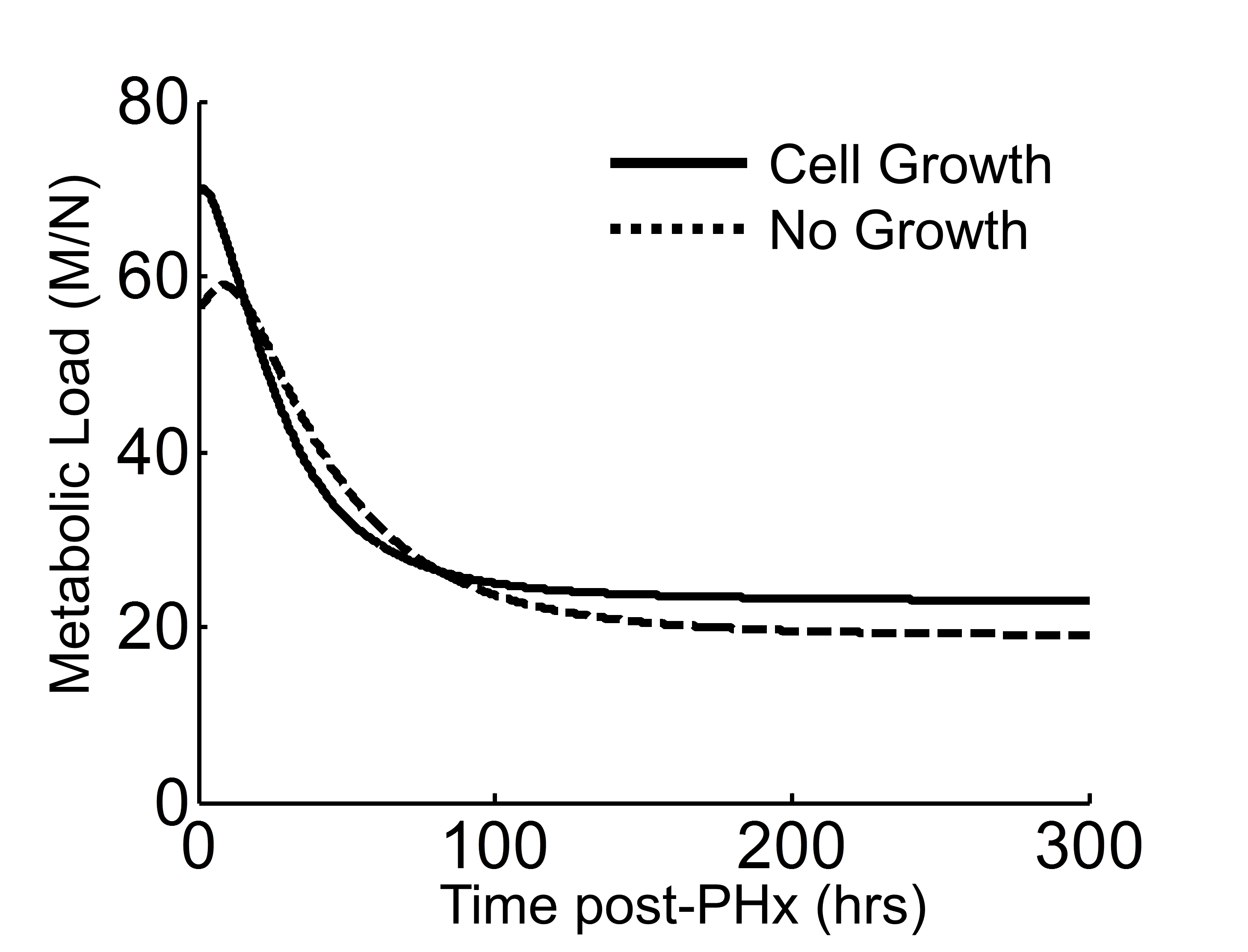

Supplement: Additional file 1: Figure S1. — Simulated metabolic load (M/N) during liver regeneration following 70 % PHx in rats for conditions including cell growth and without cell growth. (TIFF 60 kb) [file 12918_2015_220_MOESM1_ESM.tiff]

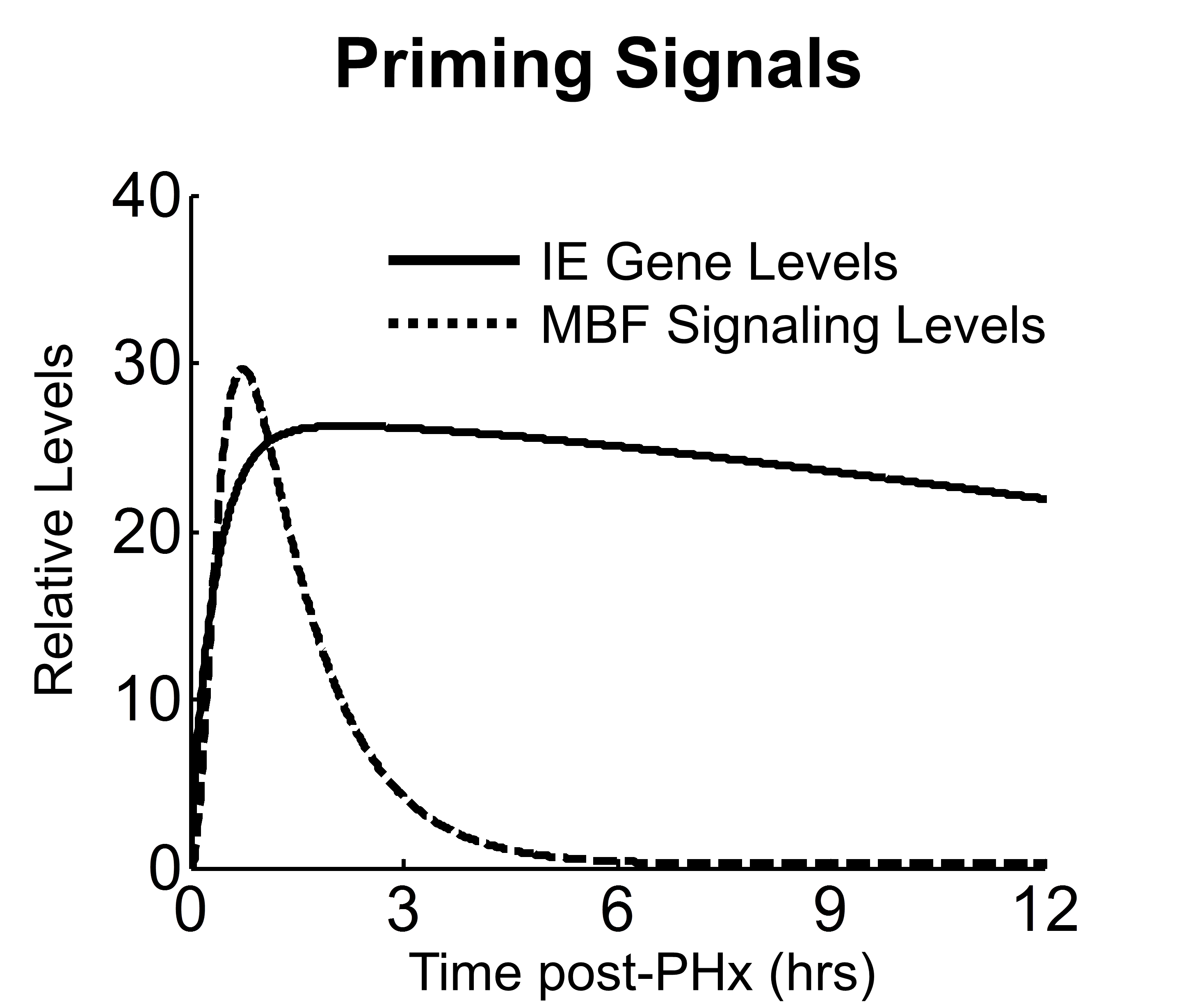

Supplement: Additional file 3: Figure S2. — Relative levels of IE genes (Fold Change) and signals released from the ECM (Relative Amount) during the priming phase. Model parameters were scaled in such a way that priming signals from Kupffer cells and from initially matrix-bound factors had relatively equal contributions to hepatocyte priming. The peak of MBF signaling occurred at approximately 45 min post-PHx. (TIFF 76 kb) [file 12918_2015_220_MOESM3_ESM.tiff]

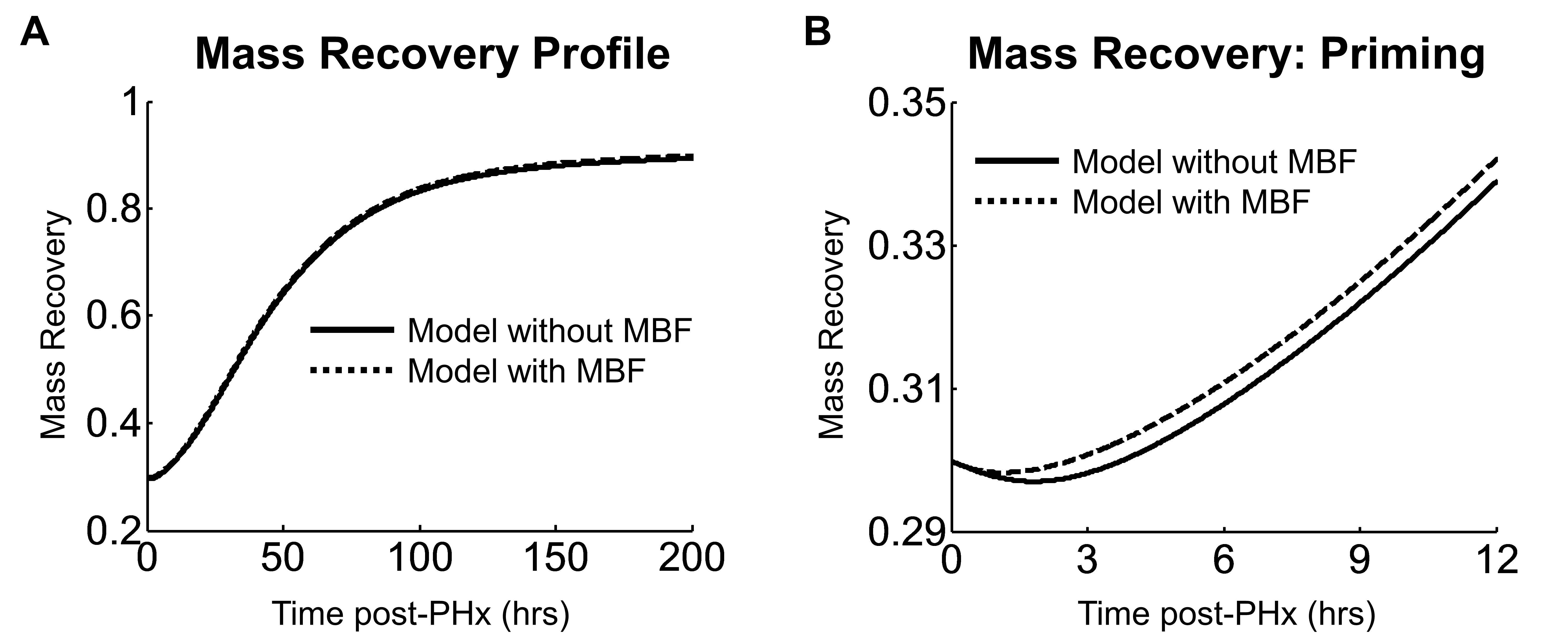

Supplement: Additional file 4: Figure S3. — Comparison of simulated mass recovery with and without considering Matrix Bound Factors (MBF). (A) Including EBF signaling in the computational model did not significantly change the overall dynamic mass recovery profile. (B) Including EBF signaling impacted the onset timing of regeneration leading to a small offset between dynamic regeneration including and excluding EBF signaling. This small offset (~0.006) remained throughout regeneration. (TIFF 1021 kb) [file 12918_2015_220_MOESM4_ESM.tif]

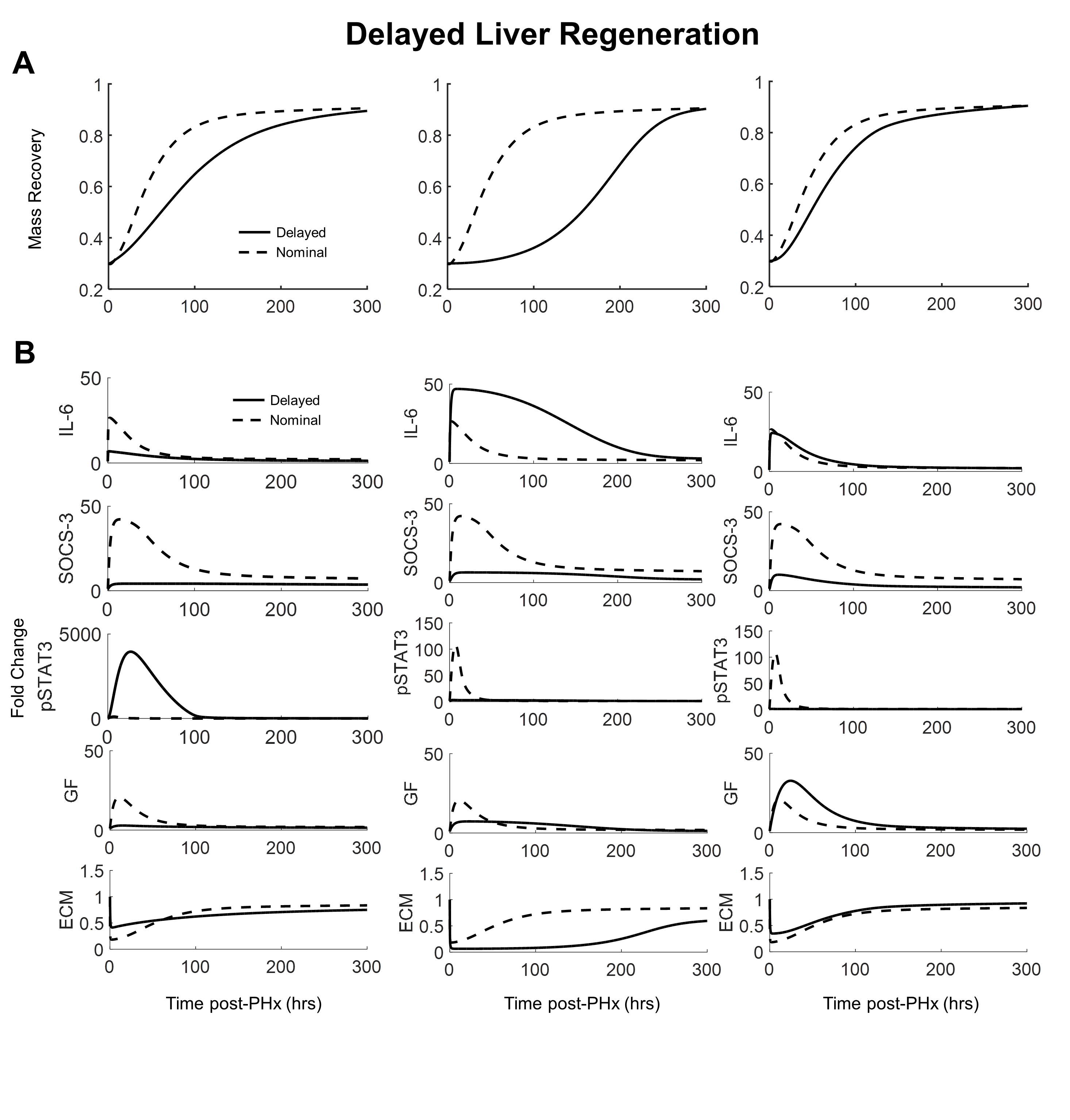

Supplement: Additional file 5: Figure S4. — Model-predicted molecular regulation profiles underlying delayed regeneration response. (A) Mass recovery of specific delayed cases of liver regeneration compared to nominal and (B) molecular regulation for each regeneration profile. Deficiencies in either priming signals or growth factor bioavailability can lead to delayed regeneration. Note that parameters related to hepatocyte response to these signals change in addition to parameters governing molecular regulation. Dashed line represents nominal profile, black line represents the profile corresponding to delayed regeneration. (TIFF 429 kb) [file 12918_2015_220_MOESM5_ESM.tif]

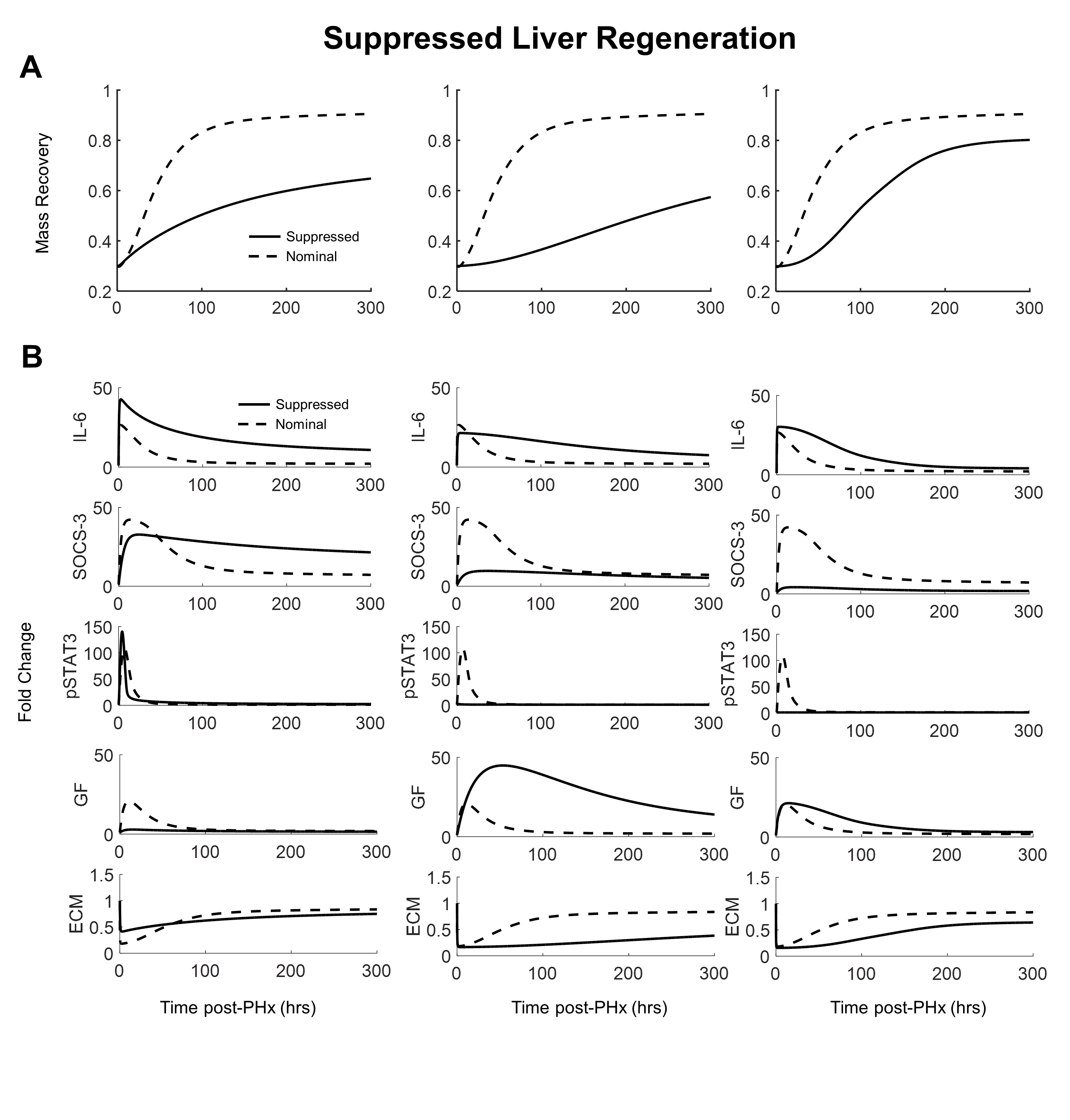

Supplement: Additional file 6: Figure S5. — Model-predicted molecular regulation profiles underlying suppressed regeneration response. (A) Mass recovery of specific suppressed cases of liver regeneration compared to nominal and (B) molecular regulation for each regeneration profile. Suppressed regeneration can be caused by deficient priming signals or deficient growth factor bioavailability. Dashed line represents nominal profile, black line represents the profile corresponding to suppressed regeneration. (TIFF 426 kb) [file 12918_2015_220_MOESM6_ESM.tif]

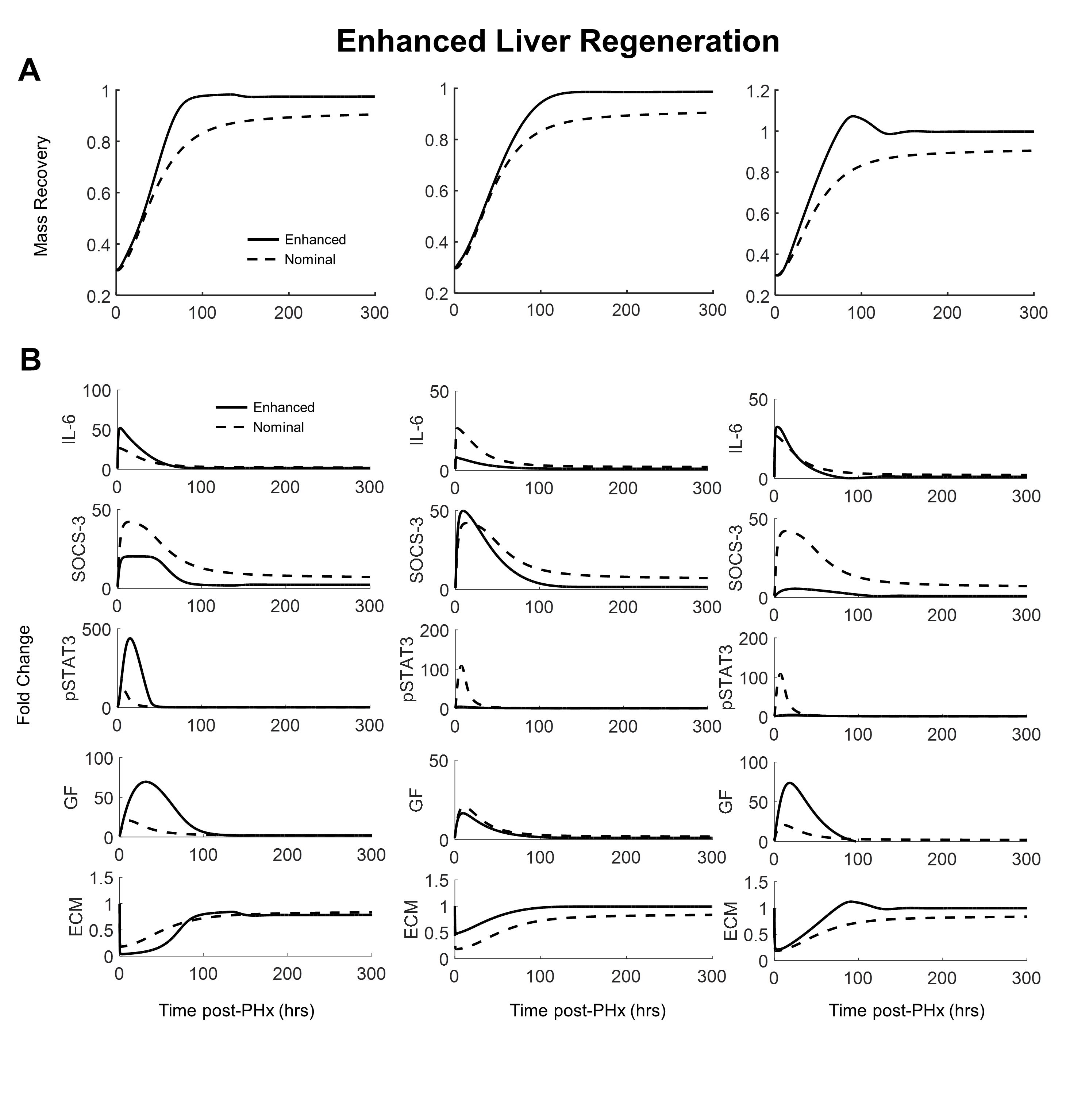

Supplement: Additional file 7: Figure S6. — Model-predicted molecular regulation profiles underlying enhanced regeneration response. (A) Mass recovery of specific enhanced cases of liver regeneration compared to nominal and (B) molecular regulation for each regeneration profile. Enhanced regeneration can be regulated by enhanced GF bioavalability or by enhanced hepatocyte response to the presence of growth factors. Dashed line represents nominal profile, black line represents the profile corresponding to enhanced regeneration. (TIFF 449 kb) [file 12918_2015_220_MOESM7_ESM.tif]

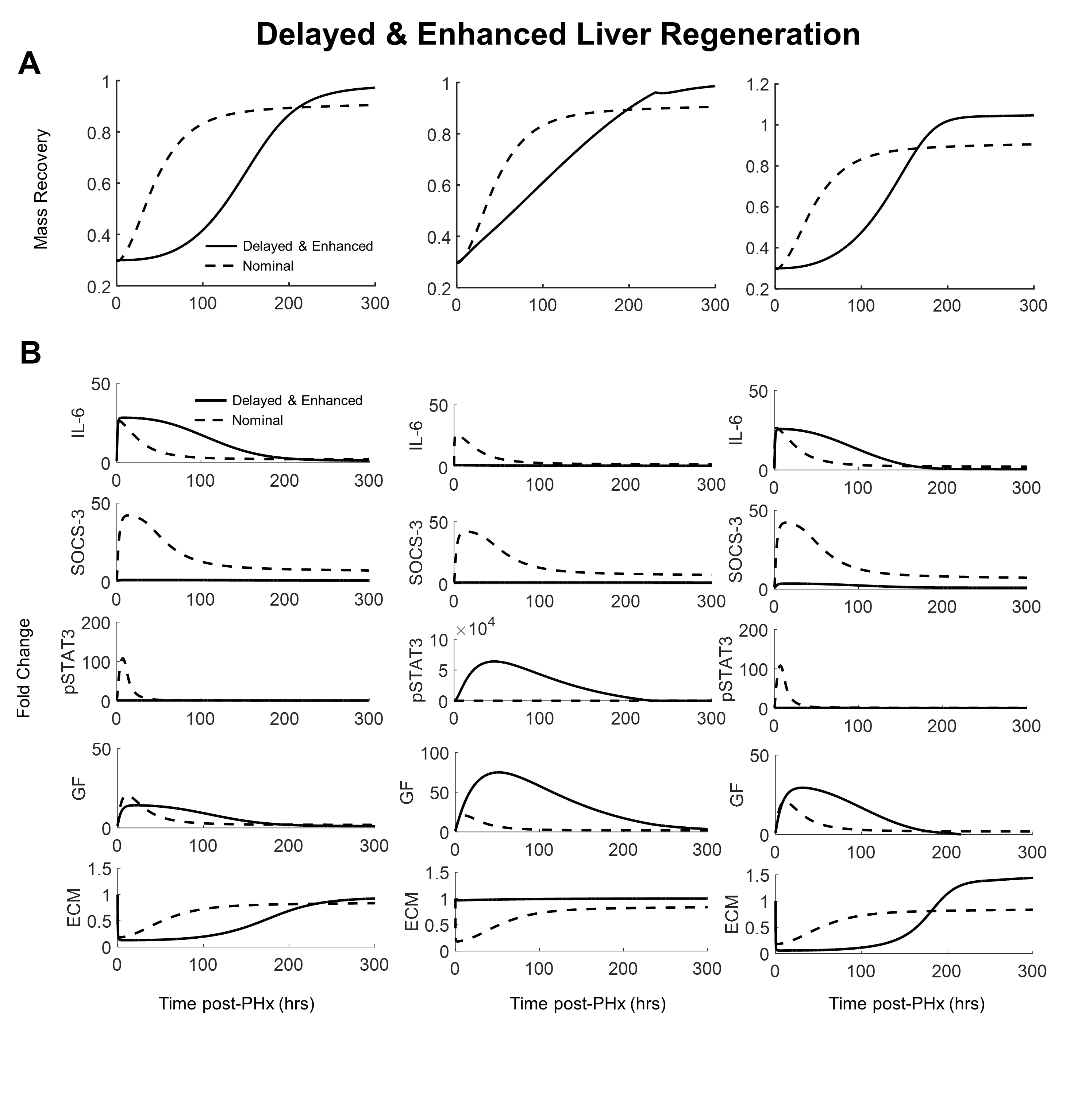

Supplement: Additional file 8: Figure S7. — Model-predicted molecular regulation profiles underlying delayed and enhanced regeneration response. (A) Mass recovery of specific delayed and enhanced cases of liver regeneration compared to nominal and (B) molecular regulation for each regeneration profile. One model-predicted cause of this profile is deficient priming signals coupled with enhanced growth factor bioavailability. Dashed line represents nominal profile, black line represents the profile corresponding to delayed and enhanced regeneration. (TIFF 437 kb) [file 12918_2015_220_MOESM8_ESM.tif]

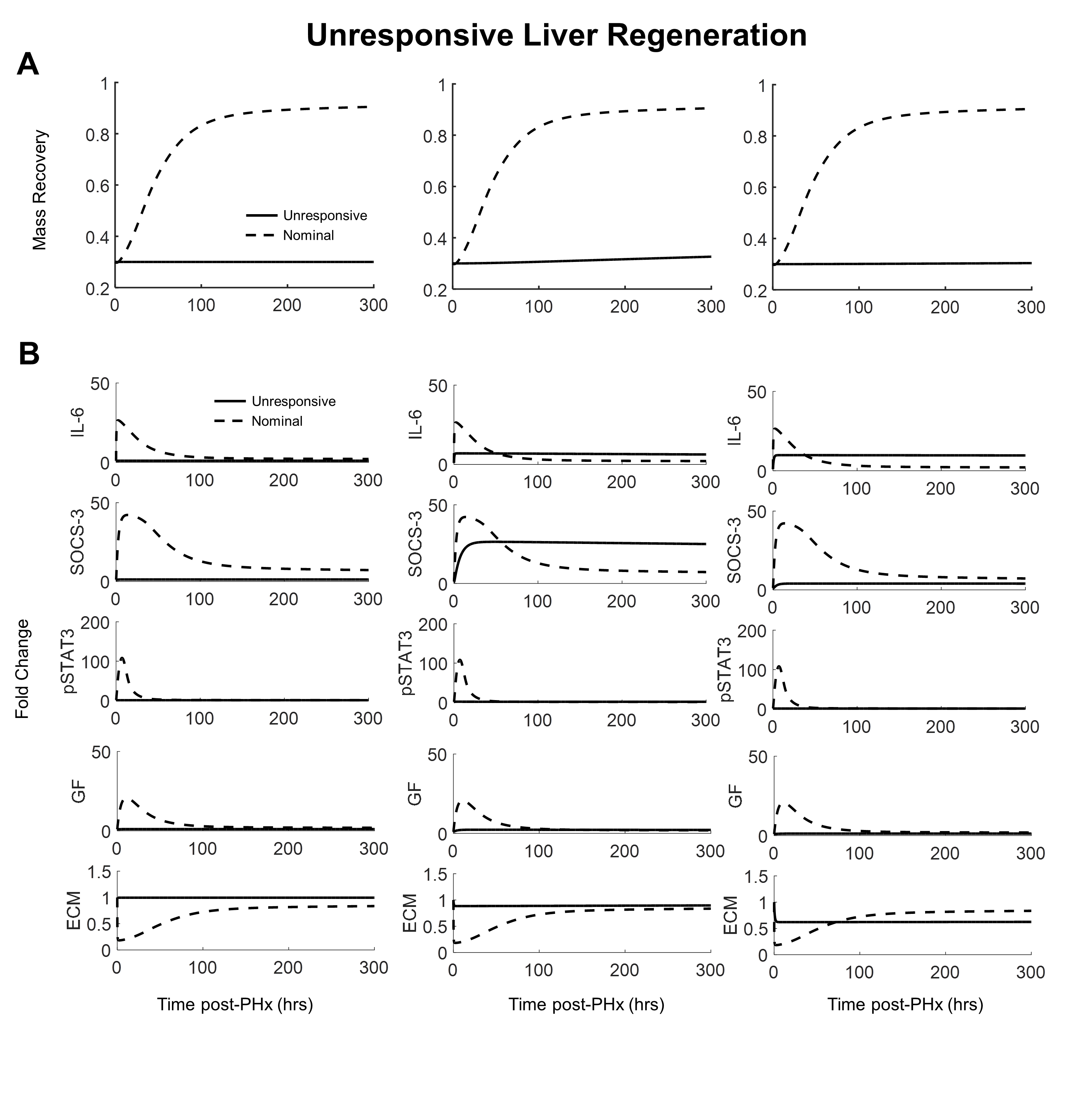

Supplement: Additional file 9: Figure S8. — Model-predicted molecular regulation profiles underlying unresponsive regeneration response. (A) Mass recovery of specific unresponsive cases of liver regeneration compared to nominal and (B) molecular regulation for each regeneration profile. Both regeneration profiles and molecular regulation appear to be unresponsive in the profiles investigated. Dashed line represents nominal profile, black line represents the profile corresponding to unresponsive regeneration. (TIFF 392 kb) [file 12918_2015_220_MOESM9_ESM.tif]

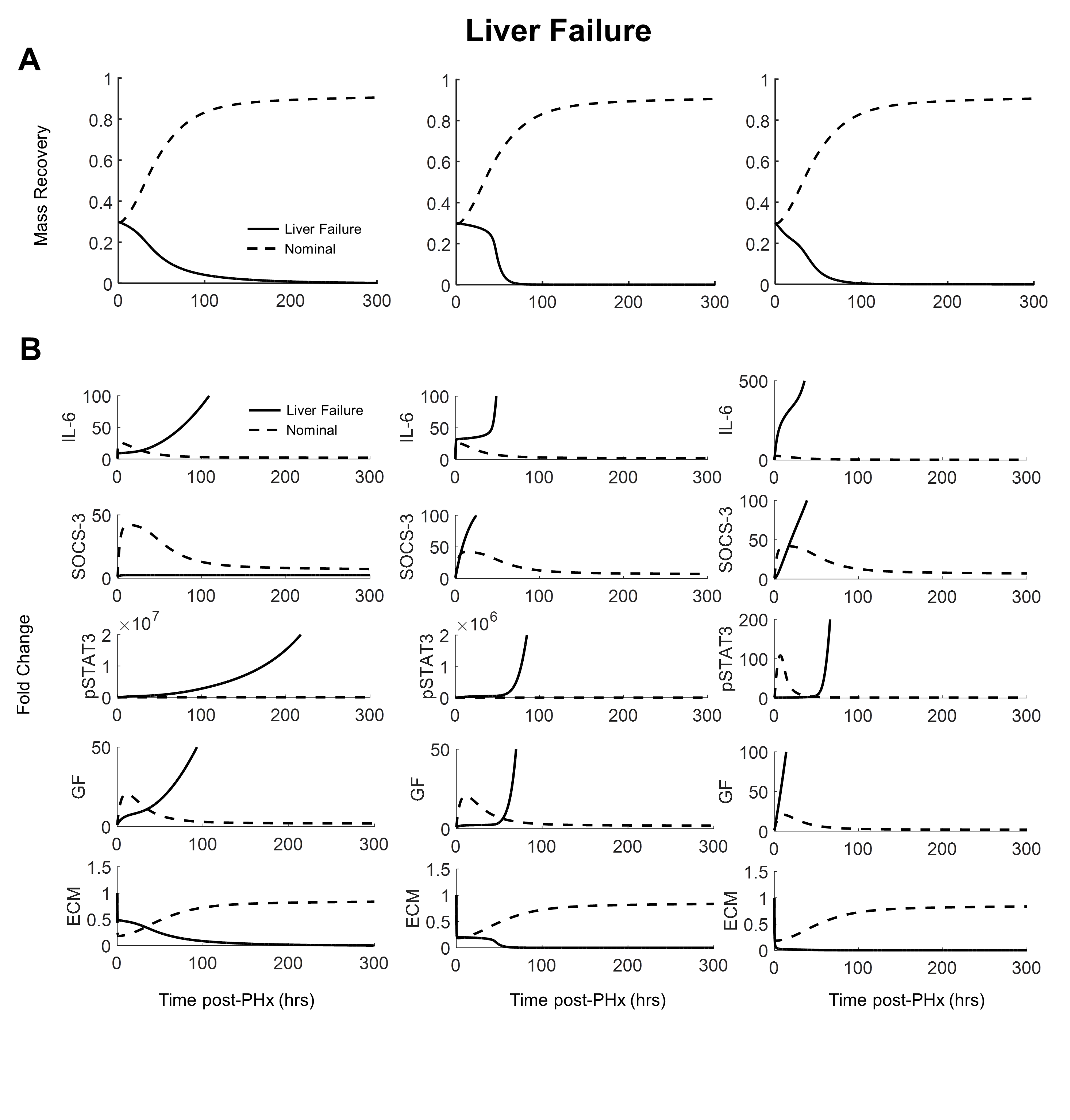

Supplement: Additional file 10: Figure S9. — Model-predicted molecular regulation profiles underlying liver failure. (A) Mass recovery of specific liver cases of liver failure compared to nominal regeneration and (B) molecular regulation for each liver failure profile. As the liver fails, Kupffer cells and hepatic stellate cells attempt to rescue the liver by producing more pro-regenerative factors. Dashed line represents nominal profile, black line represents the profile corresponding to liver failure. (TIFF 420 kb) [file 12918_2015_220_MOESM10_ESM.tif]

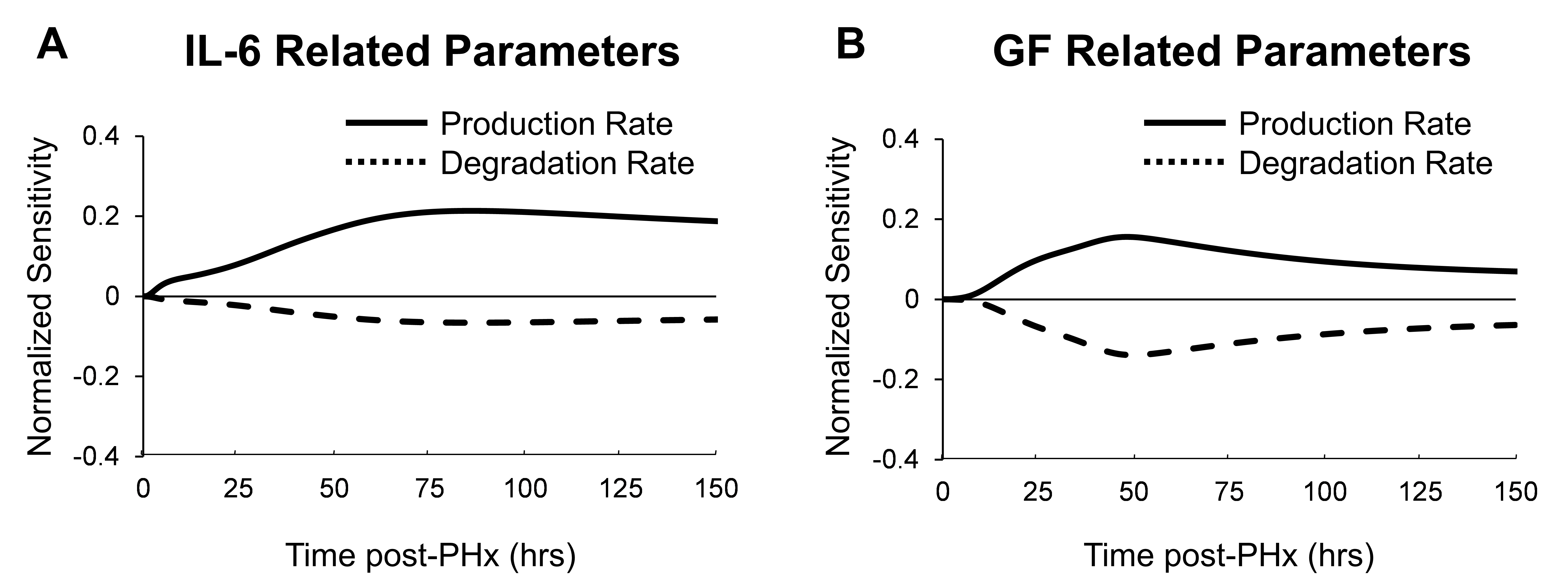

Supplement: Additional file 11: Figure S10. — Potential antagonism between pairs of parameters. We used a local sensitivity analysis to identify pairs of parameters that impact the levels of a single factor or are closely related but have opposing effects on overall mass recovery. In addition to metabolic demand and growth rate (Fig. 5c) this analysis identified (A) IL-6 production and degradation and (B) GF production and degradation as potentially antagonistic parameter pairs. (TIFF 965 kb) [file 12918_2015_220_MOESM11_ESM.tif]

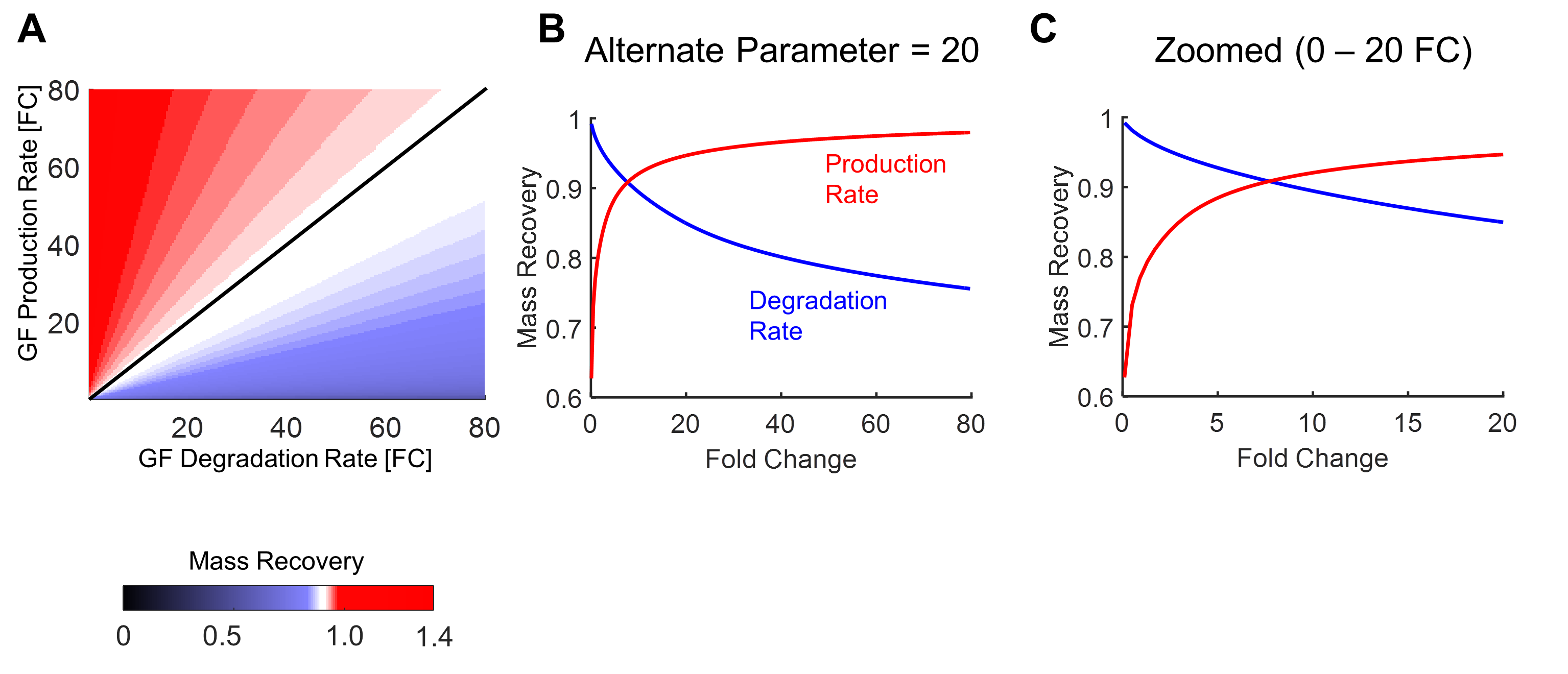

Supplement: Additional file 12: Figure S11. — Heatmap comparison of overall liver mass recovered for altered GF production and degradation rates. (A) Heatmap with rescaled color mapping showing a subtle effect of coarse-grained control of regeneration by GF production and fine-tuned control by GF degradation (legend below), (B) Mass recovery holding one parameter constant at 20 and varying the other, and (C) Mass recovery holding one parameter constant at 20 and varying the other between 0–20. All parameter changes are displayed in Fold change [FC] over nominal parameter value. The black line indicates when GF production rate fold change equals GF degradation rate fold change. Asymmetry around this line, as in (A), indicates a differential effect of GF production and degradation. (TIFF 235 kb) [file 12918_2015_220_MOESM12_ESM.tif]

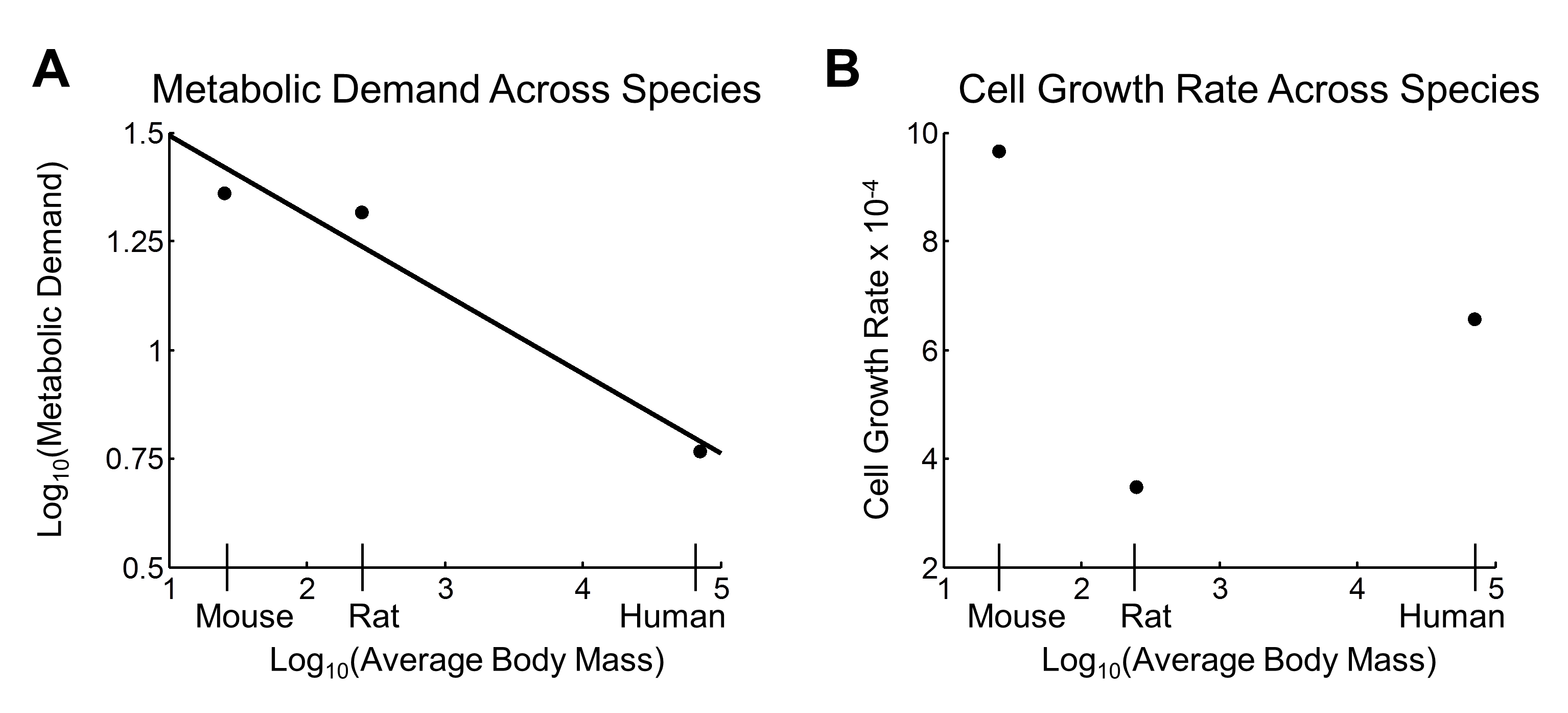

Supplement: Additional file 14: Figure S12. — Relationships between fitted parameters and body mass across species. (A) Metabolic demand shows a negative exponential relationship with body mass following the equation: Metabolic Demand = 47.315 ∗ Mass − 0.1825 (R2 = 0.95). (B) Cell growth rate for humans was estimated as the average growth for mouse and rat because there is little difference in cultured hepatocyte growth rates between species. (TIFF 66 kb) [file 12918_2015_220_MOESM14_ESM.tiff]

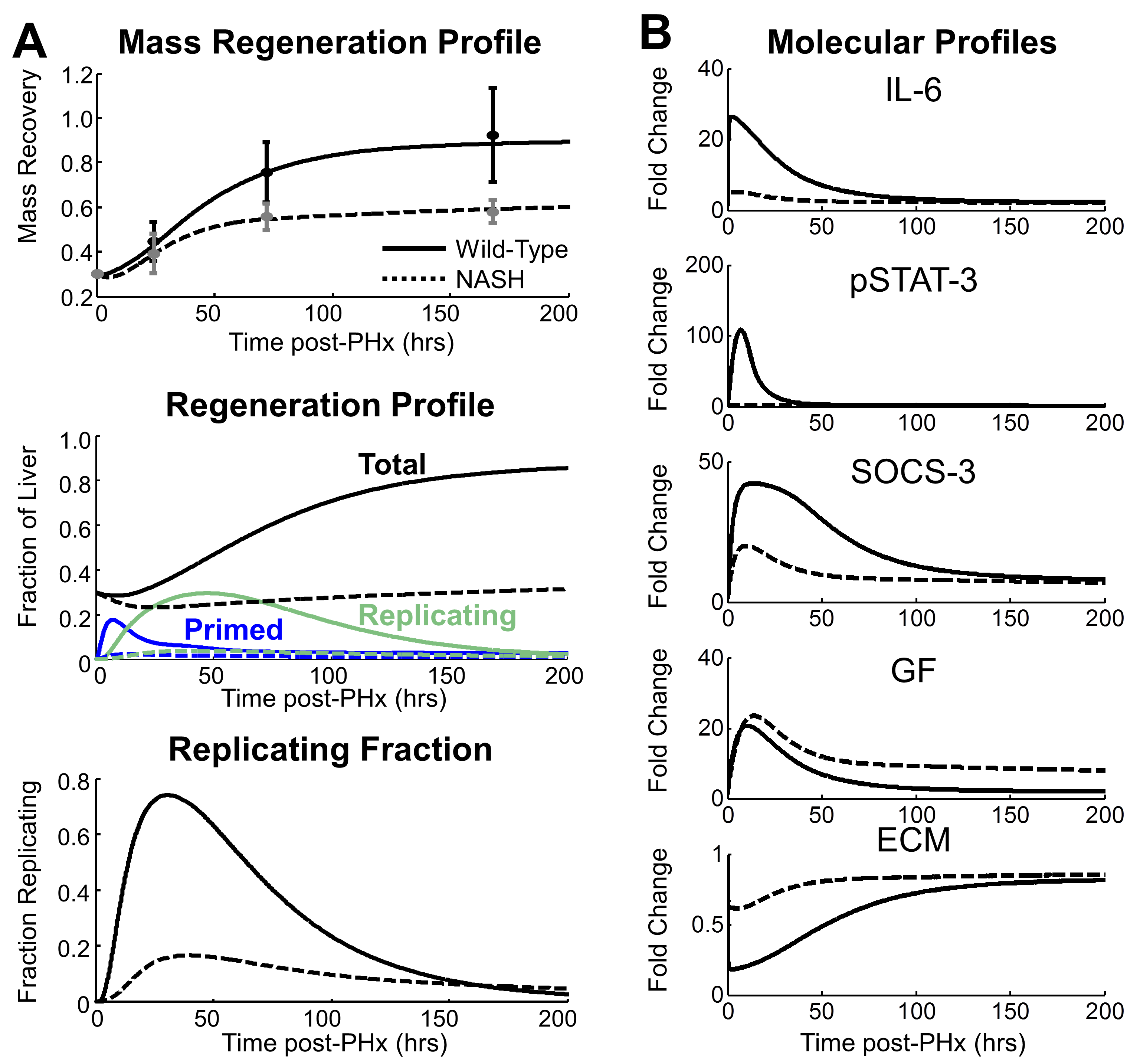

Supplement: Additional file 16: Figure S13. — Regeneration profiles for healthy livers and those with high fat, fructose-induced steatosis. (A) NASH causes an inhibited replication response following PHx. The majority of mass recovery is caused by cell growth rather than replication. (B) This regeneration profile is driven by a lack of inflammatory signaling, leading to reduced priming. Although GFs are available, the low priming means that few hepatocytes are available to enter the replication stage. (MSE = 2.25x10^−4^). (TIFF 302 kb) [file 12918_2015_220_MOESM16_ESM.tiff]

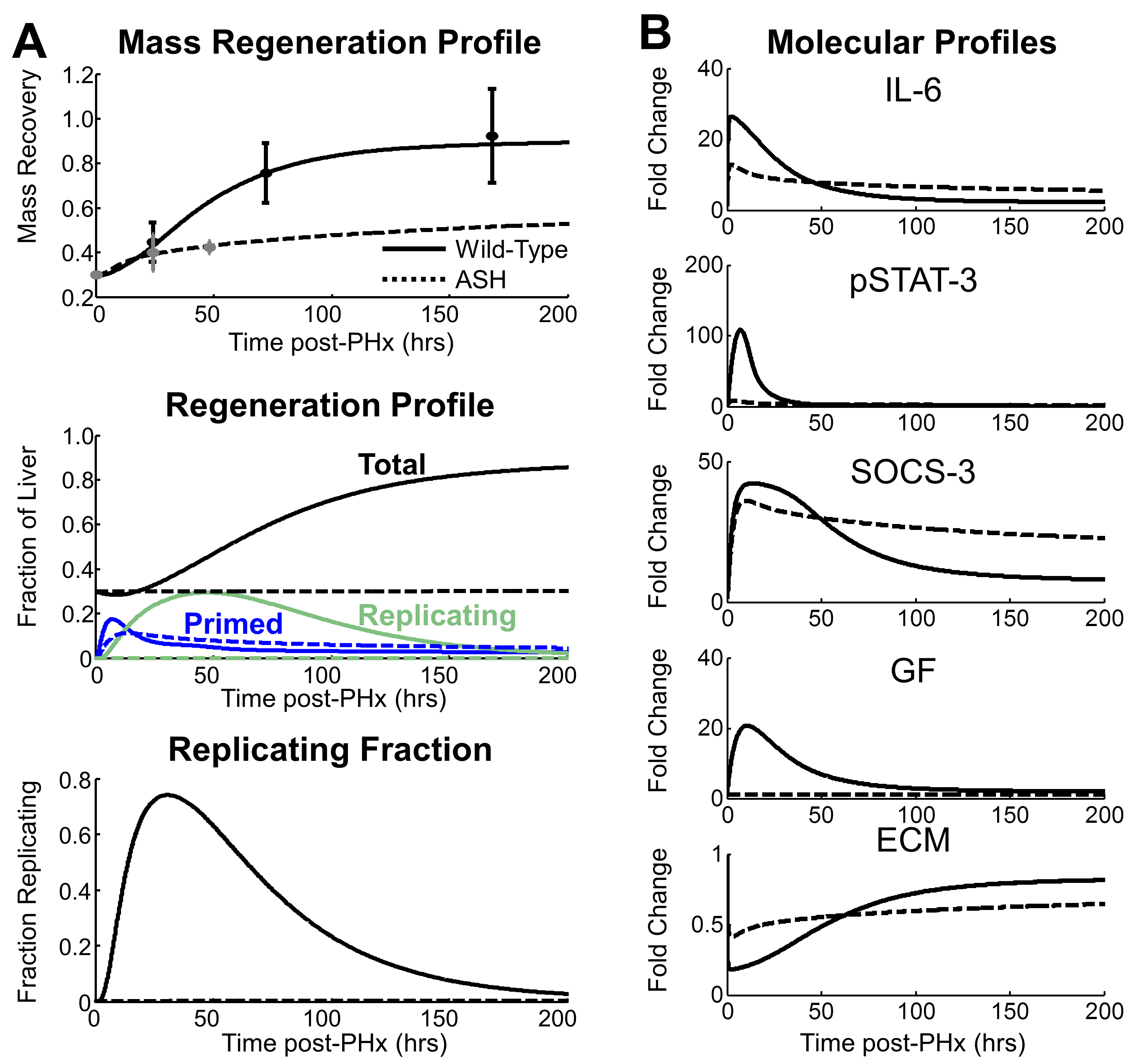

Supplement: Additional file 17: Figure S14. — Regeneration profiles for healthy livers and those with alcohol-induced steatosis. (A) Alcoholic steatosis causes suppressed liver regeneration, with little mass recovery. The slight increase in liver mass is caused predominantly by cell growth rather than replication. (B) This profile is driven by sustained inflammatory signaling, lack of growth factor bioavailability, and increased matrix deposition following wounding. (MSE = 8.82x10^−5^) (TIFF 294 kb) [file 12918_2015_220_MOESM17_ESM.tiff]

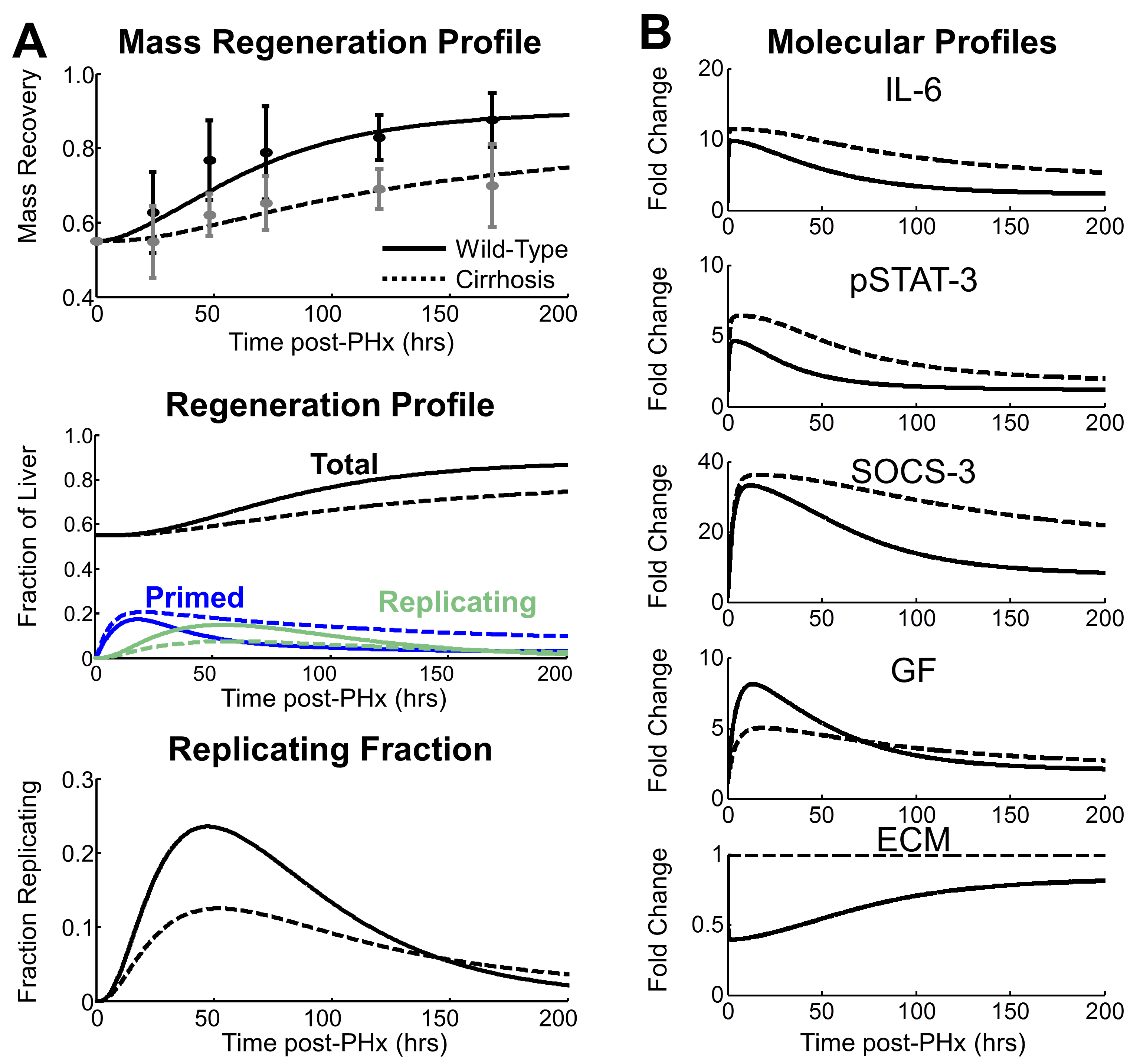

Supplement: Additional file 18: Figure S15. — Regeneration profiles for healthy livers and those with toxin-induced cirrhosis. (A) Fibrosis causes a delay in the initiation of regeneration (note change in time scale) but ultimately little change in overall mass recovery. Mass recovery is due mainly to hepatocyte replication rather than cell growth. (B) This profile is driven by a sustained inflammatory response, a lack of growth factor bioavailability, and impaired matrix deposition following wounding. (MSE = 2.50x10^−3^) (TIFF 302 kb) [file 12918_2015_220_MOESM18_ESM.tiff]

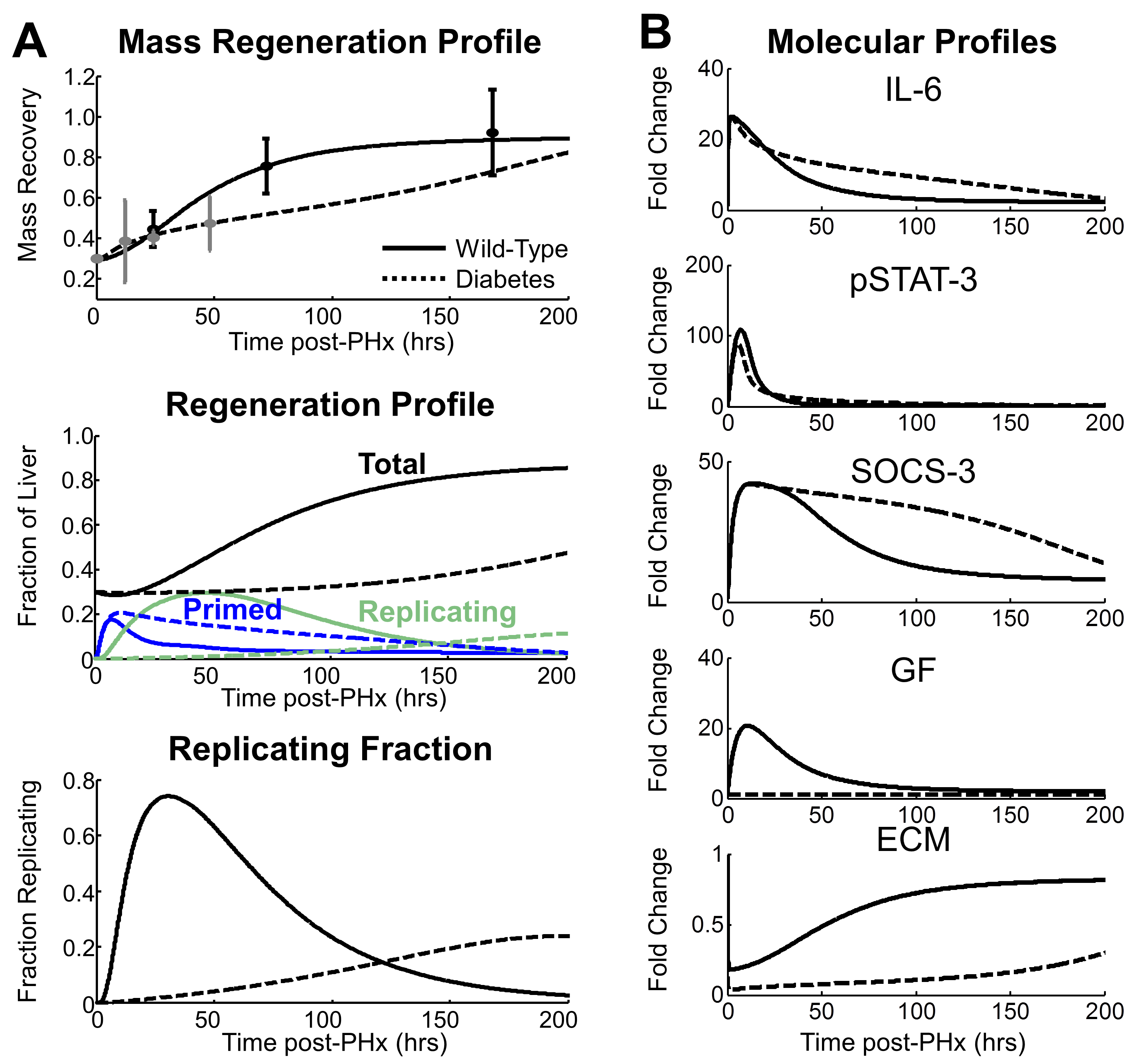

Supplement: Additional file 19: Figure S16. — Regeneration profiles for healthy livers and those with diabetes. (A) Diabetes causes enhanced priming but delayed proliferation and delayed mass recovery. (B) This profile is driven predominantly by sustained cytokine signaling and a lack of growth factor bioavailability. (MSE = 4.05x10^−4^) (TIFF 307 kb) [file 12918_2015_220_MOESM19_ESM.tiff]

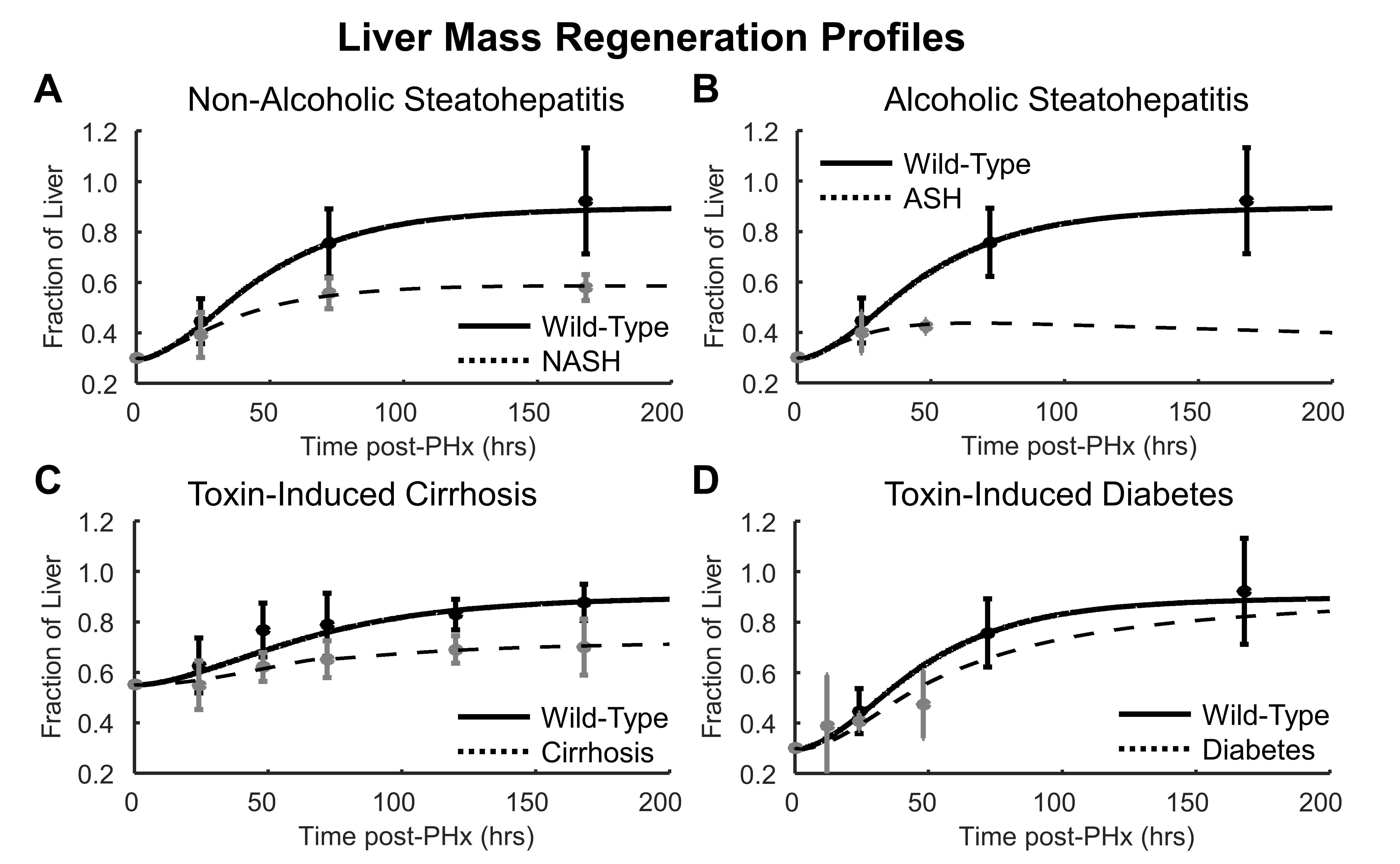

Supplement: Additional file 21: Figure S17. — Simulations using hepaotyce-specific parameter alterations compared to disease regeneration profiles. Model fits to disease regeneration profiles reveals altered hepatocyte response to non-parenchymal cell signaling is sufficient to explain disease-induced inhibition of regeneration in (A) Non-alcoholic steatohepatitis (MSE = 1.96x10^−2^), (B) Alcoholic steatohepatitis (MSE = 1.89x10^−2^), and (C) Chirrhosis (MSE = 5.14x10^−2^), but not in (D) Diabetes (MSE = 1.19). In all cases, the previous set of parameters (Additional file 15: Table S2) gave lower MSE than the hepatocyte-specific parameter alterations (Additional file 20: Table S3). MSE = Mean Squared Error between experimental and simulated data. (TIFF 1791 kb) [file 12918_2015_220_MOESM21_ESM.tif]
