# Supplementary material for: Systems analysis of non-parenchymal cell modulation of liver repair across multiple regeneration modes
Source: BMC Syst Biol. 2015 Oct 22;9:71. doi: 10.1186/s12918-015-0220-9 (PMC4618752; doi:10.1186/s12918-015-0220-9)
Supplement: Additional file 13: Table S1. — Parameter changes to simulate regeneration in multiple species (DOCX 11 kb) [file 12918_2015_220_MOESM13_ESM.docx]

**Table S1: Parameter changes to simulate regeneration in multiple species**

| Parameter | Rat | Mouse | Human |
| --- | --- | --- | --- |
| M | 20.8217 | 23.0294 | 5.8507 |
| G | 3.4742x10^-4^ | 9.6607x10^-4^ | 6.5675x10^-4^ |
