# Supplementary material for: Systems analysis of non-parenchymal cell modulation of liver repair across multiple regeneration modes
Source: BMC Syst Biol. 2015 Oct 22;9:71. doi: 10.1186/s12918-015-0220-9 (PMC4618752; doi:10.1186/s12918-015-0220-9)
Supplement: Additional file 15: Table S2. — Parameter changes to simulate alternate regeneration conditions. (DOCX 13 kb) [file 12918_2015_220_MOESM15_ESM.docx]

**Table S2: Parameter changes to simulate alternate regeneration conditions**

| Parameter | NASH | ASH | Cirrhosis | Diabetes |
| --- | --- | --- | --- | --- |
| M | 23.1645 | 14.4017 | 18.0454 | 16.4539 |
| G | 25x10^-4^ | 14x10^-4^ | 4.358x10^-4^ | 8.92x10^-4^ |
| k_IL6_ | 0.3095 | 0.7900 | 2.1565 | 1.5892 |
| κ_IL6_ | 2.3633 | 0.2073 | 1.0430 | 0.03699 |
| k_deg_ | 1.7312 | 10.5646 | 0 | 6.4571 |
| κ_ECM_ | 9.5395 | 77.7923 | 83.5649 | 1.8271x10^-6^ |
| k_GF_ | 0.0793 | 0.0002 | 0.0690 | 1.7519x10^-7^ |
| κ_GF_ | 0.1679 | 0.1196 | 0.2456 | 0.5044 |
| k_up_ | 0.0075 | 0.0071 | 0.0027 | 0.1218 |
