# Supplementary material for: Systems analysis of non-parenchymal cell modulation of liver repair across multiple regeneration modes
Source: BMC Syst Biol. 2015 Oct 22;9:71. doi: 10.1186/s12918-015-0220-9 (PMC4618752; doi:10.1186/s12918-015-0220-9)
Supplement: Additional file 20: Table S3. — Hepatocyte-specific parameter changes to simulate alternate regeneration conditions. (DOCX 12 kb) [file 12918_2015_220_MOESM20_ESM.docx]

**Table S3: Hepatocyte-specific parameter changes to simulate alternate regeneration conditions**

| Parameter | NASH | ASH | Cirrhosis | Diabetes |
| --- | --- | --- | --- | --- |
| V_JAK_ | 4.28x10^4^ | 2.50x10^4^ | 3.94x10^3^ | 7.33x10^4^ |
| K_M_^JAK^ | 4.35x10^0^ | 0.99 | 609.5 | 1.05x10^4^ |
| κ_JAK_ | 0.55 | 1.02 | 0.46 | 0.69 |
| [STAT3] | 2.54 | 4.00 | 4.18 | 1.67 |
| V_ST3_ | 723.5 | 443.4 | 790.7 | 3.5 |
| K_M_^ST3^ | 0.35 | 0.45 | 0.56 | 5.03x10^-3^ |
| κ_ST3_ | 0.04 | 0.14 | 1.9x10^-5^ | 0.17 |
| V_SOCS3_ | 3.14x10^4^ | 2.14x10^4^ | 2.26x10^4^ | 1.53x10^4^ |
| K_M_^SOCS3^ | 3.21x10^-4^ | 1.54x10^-4^ | 11.55x10^-4^ | 8.56x10^-4^ |
| κ_SOCS3_ | 1.0x10^-3^ | 2.5x10^-10^ | 0.67 | 0.12 |
| K_I_^SOCS3^ | 0.015 | 0.010 | 0.007 | 0.004 |
| V_IE_ | 255.7 | 309.7 | 20.6 | 364.7 |
| K_M_^IE^ | 43.2 | 28.5 | 28.6 | 0.53 |
| κ_IE_ | 6.8 | 6.9 | 8.2 | 1.8 |
